# Clinical Pharmacology in Sarcoidosis: How to Use and Monitor Sarcoidosis Medications

**DOI:** 10.3390/jcm13051250

**Published:** 2024-02-22

**Authors:** Sooyeon Kwon, Marc A. Judson

**Affiliations:** 1 Samuel S. Stratton Veterans Affairs Medical Center, Albany, NY 12208, USA; 2 Division of Pulmonary and Critical Care Medicine, Albany Medical College, Albany, NY 12208, USA; judsonm@amc.edu

**Keywords:** sarcoidosis, pharmacotherapy, corticosteroid, biologics, DMARD

## Abstract

When sarcoidosis needs treatment, pharmacotherapy is usually required. Although glucocorticoids work reliably and relatively quickly for sarcoidosis, these drugs are associated with numerous significant side effects. Such side effects are common in sarcoidosis patients, as the disease frequently has a chronic course and glucocorticoid treatment courses are often prolonged. For these reasons, corticosteroid-sparing and corticosteroid-replacing therapies are often required for sarcoidosis. Unfortunately, many healthcare providers who care for sarcoidosis patients are not familiar with the use of these agents. In this manuscript, we provide a review of the pharmacotherapy of sarcoidosis. We discuss the mechanism of action, dosing, side-effect profile, approach to monitoring and patient counselling concerning glucocorticoids, and the common alternative drugs recommended for use in the recent European Respiratory Society (Lausanne, Switzerland) Sarcoidosis Treatment Guidelines. We also discuss the use of these agents in special situations including hepatic insufficiency, renal insufficiency, pregnancy, breastfeeding, vaccination, and drug–drug interactions. It is hoped that this manuscript will provide valuable practical guidance to clinicians who care for sarcoidosis patients.

## 1. Introduction

Sarcoidosis is a multisystem granulomatous disease of unknown cause. Sarcoidosis is usually treated with pharmacotherapy. The treatment of sarcoidosis is nuanced because the disease outcome varies from an asymptomatic state to a life-threatening disease, therapeutic agents are associated with significant toxicity, the prognosis of the disease is problematic to predict, and the effectiveness of specific drugs is dependent upon the specific organs involved. All these issues are discussed in detail in the recent European Respiratory Society (ERS) Clinical Practice Sarcoidosis Treatment Guidelines [1].

The ERS guidelines review the indications for numerous drugs used for the treatment of sarcoidosis. Many healthcare providers who care for sarcoidosis patients are not familiar with the use of these agents. Many are primary care physicians or subspecialists such as pulmonologists, ophthalmologists, and dermatologists who are unaccustomed to using many of these drugs in their routine practice. In this manuscript, we review characteristics of the pharmacologic agents that are most often used for the treatment of sarcoidosis. We will focus on the proper use and monitoring of these pharmacologic agents in clinical practice.

## 2. Glucocorticoids

### 2.1. Mechanism of Action

There are numerous mechanisms responsible for the anti-inflammatory effects of glucocorticoids including its inhibitory effects on a vast number of mediators such as tumor necrosis factor alpha (TNFa), various interleukins (IL), endothelial leukocyte adhesion molecule 1 (ELAM-1), and intercellular adhesion molecule 1 (ICAM-1), which are impaired by glucocorticoids [2].

### 2.2. General Treatment Indications for Glucocorticoids in Sarcoidosis

Glucocorticoids are considered the drug of choice for most forms of sarcoidosis [3]. These drugs are recommended as first-line agents for pulmonary, skin, cardiac, and neurologic sarcoidosis in the European Respiratory Society (ERS) Clinical Practice Sarcoidosis Treatment Guidelines [1]. However, because of the myriad of potential side effects from glucocorticoids, several other drugs are considered for the treatment of sarcoidosis for their glucocorticoid-sparing or glucocorticoid-replacing effects [3].

### 2.3. Dosing

Glucocorticoid dosing in sarcoidosis is not standardized. For symptomatic pulmonary sarcoidosis, the recent European Respiratory Society (ERS) Sarcoidosis Treatment Guidelines recommend an initial daily prednisone dose of 20 mg. However, glucocorticoid dosing in sarcoidosis varies based on the severity of disease, the organ involved, the risk of glucocorticoid side effects, the risk of leaving sarcoidosis partially treated or untreated, and the efficacy of concomitant corticosteroid-sparing medications. Various glucocorticoid preparations and potencies are listed in Table 1.

Although glucocorticoids are primarily metabolized in the liver as they are substrates for CYP3A4, hepatic dose adjustment is not required. Renal dose adjustment is also not required.

### 2.4. Side Effects and Monitoring

Glucocorticoids have numerous side effects such as gastritis, weight gain, hypertension, fluid retention, hyperglycemia, skin atrophy, impaired wound healing, osteoporosis, depression, mood change, adrenocortical insufficiency (when glucocorticoids are tapered or withdrawn), Cushing syndrome, decreased growth in children, myopathy, glaucoma, cataract, and an increased risk of infection. The risk for developing these side effects is dependent upon individual patient factors such as comorbidities. Clinicians may alter glucocorticoid regimens based on individual patient risks. Glucocorticoid side effects are also dose dependent, and it is recommended to use the smallest dose for the shortest duration possible. In the case of sarcoidosis, which is often a chronic condition, glucocorticoid-sparing agents should be considered in patients requiring glucocorticoid therapy for more than a few months [5,6].

Some glucocorticoid side effects can be detected by eliciting symptoms from the patient such as gastrointestinal discomfort or mood change. However, many glucocorticoid side effects may not be detected at an early stage because they do not result in appreciable symptoms; therefore, patients receiving glucocorticoids must be monitored for the development of potential side effects while they are asymptomatic. The developments of weight gain, hypertension, hyperlipidemia, and diabetes (components of the metabolic syndrome) are serious glucocorticoid complications for which the patient should regularly be evaluated. Side effects that the patient cannot easily perceive, such as osteoporosis and eye disease, need to be regularly monitored. Table 2 lists parameters that should be assessed at the initiation of glucocorticoid therapy as well as those that should be monitored during therapy.

The American College of Rheumatology guidelines recommend osteoporosis monitoring for all adults initiating glucocorticoid therapy or continuing glucocorticoid therapy ≥2.5 mg/day for more than three months. [7] An initial fracture-risk assessment using the clinical fracture-risk factor assessment (FRAX^®^, https://frax.shef.ac.uk/FRAX/tool.aspx, accessed on 20 November 2023) is strongly recommended for all such patients, including for those who have never had a fracture-risk assessment or have been previously treated for osteoporosis. FRAX^®^ estimates the fracture risk based on many factors including alcohol use, smoking history, hypogonadism, history of prior fractures, body weight, height, parental history of hip fracture, fall history, rheumatoid arthritis, thyroid disease, hyperparathyroidism, malabsorption, chronic liver disease, inflammatory bowel disease, and height loss. Treatment recommendations for loss of bone density are based on the FRAX^®^ score. If available, bone-mineral density (BMD) testing with vertebral-fracture assessment (VFA) or spinal x-ray is recommended as soon as possible after starting glucocorticoid therapy as a baseline measurement, and then every 1–2 years thereafter while continuing a glucocorticoid regimen [7].

### 2.5. Drug Interactions

Concomitant use of glucocorticoids and fluoroquinolones increases the risk of tendonitis and tendon rupture [12]. There are inconsistent reports regarding drug interaction between glucocorticoids and antacids; while some studies reported that concomitant antacid use may decrease glucocorticoid’s bioavailability by 40–75%, [13,14] others reported no change in bioavailability [15,16]. Careful monitoring of the international normalized ratio (INR) is required with concomitant use of warfarin and glucocorticoids, as glucocorticoids may increase the INR [17].

Glucocorticoids are metabolized in the liver via the CYP450 3A4 enzyme (CYP3A4). Therefore, concomitant use of CYP3A4 inhibitor(s) or inducer(s) may change glucocorticoid metabolism: CYP3A4 inhibitors may decrease glucocorticoid metabolism leading to increased anti-inflammatory effectiveness with an increased risk of side effects. Conversely, CYP3A4 inducers can increase prednisone metabolism, which can lead to diminished glucocorticoid effectiveness (Table 3). The effects of CYP3A4 inhibitors and inducers on glucocorticoid potency and side effects are often clinically significant [18,19,20].

### 2.6. Special Situations

*Pregnancy*: Because sarcoidosis frequently occurs in women of childbearing age, glucocorticoid use during pregnancy is a clinically relevant issue. Prednisone and methylprednisolone are non-fluorinated glucocorticoids and are therefore the preferred oral glucocorticoids during pregnancy because the placental barrier limits its transport to the fetus, while fluorinated glucocorticoids such as dexamethasone and betamethasone can readily cross the placenta [21]. Conflicting data have been reported regarding the associations between systemic glucocorticoid use during the first trimester of pregnancy and the development of cleft lip and palate as well as low birth rate [22,23]. These associations appear to be influenced by the glucocorticoid dose, duration of use, frequency, and indication for use [23,24,25]. The general recommendation for glucocorticoid use during pregnancy is to use prednisone at the lowest effective dose for shortest duration possible, and to avoid high doses, particularly during the first trimester [24,26].

*Breastfeeding*: Mothers should be counselled that glucocorticoids are present in breast milk. Although glucocorticoids are generally well tolerated by the child receiving breast milk from mothers using standard glucocorticoid doses, it is recommended to monitor the infant for adverse events such as growth suppression. The European Respiratory Society/Thoracic Society of Australia and New Zealand (ERS/TSANZ) task force team recommended waiting 3–4 h after a prednisone dose to begin breastfeeding to minimize the potential glucocorticoid exposure to the breastfeeding child [27]. Based on prednisone’s half-life, approximately 87–94% of the drug is eliminated from mother’s plasma by this time.

*Osteoporosis*: For the prevention and treatment of glucocorticoid-induced osteoporosis, the American College of Rheumatology guidelines suggest specific recommendations for adults who are taking a prednisone equivalent of ≥2.5 mg daily for >3 months, based on the individual patient’s level of risk: low risk, moderate, high, and very high risk, respectively [7]. These levels of risk are based on the glucocorticoid dose, dual-energy X-ray absorptiometry (DEXA) T score and Z score, FRAX^®^ score, and a prior history of osteoporosis-related fracture. Specific details can be found in the 2022 American College of Rheumatology Guideline for the Prevention and Treatment of Glucocorticoid-Induced Osteoporosis [7].

*Pneumocystis jirovecii pneumonia (PJP) prophylaxis: PJP* Prophylaxis with trimethoprim and sulfamethoxazole (TMP/SMX) is often used in patients receiving high-dose glucocorticoid therapy. Although there is no consensus on a specific glucocorticoid dose that requires TMP/SMX for PJP prophylaxis, most reports recommend PJP prophylaxis in patients receiving a prednisone equivalent ≥20~30 mg daily [28,29,30]. In clinical practice, PJP prophylaxis with TMP/SMX is generally not used in sarcoidosis patients unless they are receiving concomitant immunosuppressive medication.

*Glucocorticoid interaction with the QuantiFERON test:* The QuantiFERON test is an interferon gamma (IFN-γ) release assay (IGRAs) that measures an immunologic response to TB antigen exposure [31]. This test involves a positive control tube to measure IFN-γ release in blood in response to a non-specific lymphocyte activator, i.e., phytohemagglutinin [32]. High doses of glucocorticoids (≥20 mg/day of prednisone equivalent) and other immunosuppressants often cause an inadequate IFN-γ release in the phytohemagglutinin-stimulated tube, which leads to an “indeterminate” QuantiFERON test result. If an indeterminate QuantiFERON test result is obtained while the patient is receiving high-dose glucocorticoids, then a repeat QuantiFERON test is recommended after tapering glucocorticoids to <20 mg of daily prednisone [33,34]. Because of this potential effect of high-dose glucocorticoids on the QuantiFERON test result, it is prudent to perform this test prior to initiating high-dose corticosteroids in patients with severe sarcoidosis who are thought likely to be prescribed biologic therapy that requires prior latent tuberculosis (TB) infection screening.

*Vaccination*: Influenza vaccine can be administered while patients are receiving glucocorticoids at any dose. Other non-live-attenuated vaccines are recommended when the prednisone equivalent dose is <20 mg daily. However, for prednisone equivalent doses of ≥ 20 mg daily, other non-live-attenuated vaccines are recommended to be deferred until the glucocorticoid dose is tapered to <20 mg prednisone daily equivalent. For live-attenuated vaccines, glucocorticoids are recommended to be held from four weeks before until four weeks after vaccination. For patients receiving a lower dose of glucocorticoids (<20 mg prednisone equivalent), glucocorticoid therapy may be continued while the patient receives a live-attenuated vaccine [35]. The following glucocorticoid regimens may be continued while receiving a live vaccine: short-term use of glucocorticoid <14 days, low-to-moderate dose prednisone (defined as <20 mg/day or <2 mg/kg/day for a child), long-term but alternate day glucocorticoids, daily use of topical glucocorticoids, inhaled corticosteroids, and localized glucocorticoid injections into joints [36].

The Centers for Disease Control and Prevention (CDC), in January 2022, updated their recommendation on Shingrix^®^ Recombinant Zoster Vaccine (RZV) such that all individuals ≥19 years old who have an immunocompromised condition or who will imminently receive immunocompromised medication are eligible for RZV administration [37]. This recommendation applies not only to glucocorticoids but also to all other immunosuppressants that are discussed in this review.

### 2.7. Counseling Points for a Patient Receiving Glucocorticoid(s)

- Take glucocorticoids with food to prevent gastrointestinal discomfort.
- Take glucocorticoids in the morning time to minimize insomnia.
- Educate the patient concerning potential glucocorticoid side effects including hyperglycemia, osteoporosis, adrenocortical insufficiency, weight gain, fluid retention, hypertension, mood change, myopathy, glaucoma, cataract, and infections.
- Contact the healthcare provider if an infection occurs, or if an invasive procedure is planned that may increase the risk of infection. Glucocorticoids may have to be held temporarily in this instance.
- Encourage vaccination prior to initiating glucocorticoids, as vaccination is a highly effective infection mitigation strategy.
- Patients receiving glucocorticoids or another immunosuppressive medication are eligible for RZV, Shingrix^®^ (GlaxoSmithKline, Durham, NC, USA).

## 3. Methotrexate

Methotrexate (MTX) is a disease modifying anti-rheumatic drug (DMARD) that is effective for many rheumatologic and inflammatory conditions, including sarcoidosis. Originally, MTX was used for childhood leukemia in the 1940s. Placebo-controlled clinical trials in the 1980s demonstrated MTX’s effectiveness for the treatment of rheumatoid arthritis [38,39,40,41], which currently is the first-line therapy [42].

### 3.1. Mechanism of Action

MTX’s therapeutic effectiveness is achieved by inhibiting the enzyme dihydrofolate reductase (DHFR). DHFR reduces dihydrofolate to tetrahydrofolate, which is necessary during DNA synthesis.

### 3.2. General Treatment Indications for Methotrexate in Sarcoidosis

MTX is regarded as a second-line agent for sarcoidosis. MTX is specifically recommended as a second-line agent for pulmonary, skin, cardiac, and neurologic sarcoidosis in the European Respiratory Society (ERS) Clinical Practice Sarcoidosis Treatment Guidelines [1]. The drug is often effective as a glucocorticoid-sparing agent and, in approximately 25% of cases, as a glucocorticoid-replacing agent [43]. Because of drug accumulation with renal insufficiency (vide infra), MTX is not recommended for the treatment of renal sarcoidosis [44].

### 3.3. Dosing

Various MTX dosing regimens have been used for different medical conditions. We will review the clinical approach concerning the most recent and generally accepted low-dose MTX regimens for the treatment of sarcoidosis. Higher doses of MTX regimen for oncology will not be discussed in this review.

It is important to immediately stress that patients should be specifically counselled to take MTX once weekly, and not daily. Dosing error is one of the major causes of MTX overdose [45]. The usual MTX dose for the treatment of sarcoidosis is between 5 mg and 25 mg, with this total dose given once per week. The usual starting dose ranges from 5 mg to 12.5 mg, and then can be titrated up by 2.5~5 mg every 1–2 weeks to reach the desired target dose. Dosing above 25 mg per week has minimal additional benefit and is not routinely recommended [46].

The oral bioavailability of MTX is significantly reduced with oral doses of ≥15 mg per week, as there is a plateau of absorption above that dose [47]. Therefore, when an oral MTX dose above 15 mg weekly is needed, a split oral dosing strategy can be used to increase bioavailability: administer half of the weekly oral dose in the morning, and the remaining half in the evening on the same day (12 h apart). A split dose of oral MTX regimen is conditionally recommended over switching to an alternative DMARD(s) for patients not tolerating oral weekly MTX per 2021 ACR rheumatoid arthritis guidelines [42]. This approach can be extrapolated for the treatment of sarcoidosis. We acknowledge that practice varies geographically and that guidelines from other regions may not explicitly comment on the split dosing recommendation.

MTX can be administered by the subcutaneous route. Subcutaneous administration bypasses the gastrointestinal (GI) tract such that patients who have GI side effects may better tolerate the drug. Subcutaneous administration of MTX also results in improved drug bioavailability compared to the oral route. The issues of inadequate oral bioavailability for MTX doses above 15 mg can also be avoided by administering MTX by subcutaneous injection [47,48,49]. The oral-to-subcutaneous dose conversion is 1:1.

MTX is hepatically metabolized to polyglutamate MTX, which is an active metabolite. Because polyglutamate MTX is excreted renally, individuals with compromised renal function may have a high risk of side effects from accumulation of this metabolite. Therefore, when the estimated glomerular filtration rate (eGFR) is <50~60 mL/min, the MTX dose needs to be reduced appropriately (Table 4) [50]. MTX is contraindicated in patients receiving hemodialysis and peritoneal dialysis [51]. MTX is also contraindicated in patients with a chronic pleural effusion, which acts as a drug sanctuary and increases the risk of side effects [52].

### 3.4. Side Effects and Monitoring

MTX may cause folate deficiency. Folic acid at a dose of 1 mg to 4 mg daily is recommended for patients receive MTX [53]. Folic acid can prevent MTX toxicity without affecting the effectiveness of MTX. In contrast, folinic acid, an active form of folic acid also known as leucovorin, is a reduced folate that can negate the beneficial effects of MTX. Therefore, folic acid can be dosed daily, seven days per week, even on the day of the MTX dose, whereas leucovorin should be administered at least 12 h after MTX use to preserve MTX’s therapeutic effect [53].

Leucovorin is a valuable agent to rescue patients from MTX toxicity. Leucovorin may provide a significant benefit in patients who have known methylenetetrahydrofolate reductase (MTHFR) deficiency or in those have developed MTX side effects daily while receiving a high dose of folic acid supplementation (3–4 mg daily) [54].

Although MTX is usually well tolerated, gastrointestinal side effects, fatigue, headaches, and dizziness may occur. MTX is immunosuppressive and increases the risk of infection. Hepatotoxicity may occur. Reductions in blood cell lines due to bone-marrow suppression may develop and may require a reduction of the MTX dose or discontinuing the drug if bone-marrow suppression is severe. Folic acid supplementation can mitigate these MTX toxicities. Therefore, folic acid should be prescribed along with MTX.

Blood labs such as complete blood count (CBC), serum renal function tests, serum liver function tests, and viral hepatitis serologies are recommended prior to initiation of MTX. CBC, renal, and hepatic function tests are required frequently as often as every two to four weeks initially for at least the first two to three months and every three months thereafter [55].

Patients receiving MTX should have their mean corpuscular volume (MCV) monitored, as it may be an early sign of MTX-induced vitamin B12 or folate deficiency. However, a high MCV is not an indication to adjust the MTX dose if the blood cell lines are not significantly reduced. When significant bone-marrow suppression develops, leucovorin rescue therapy and switching to an alternative drug should be considered.

MTX rarely causes interstitial lung disease. A persistent cough and unexplained dyspnea may be the first symptoms of this complication. A baseline chest radiograph is recommended as it may be used for comparison if MTX pulmonary toxicity is eventually considered [56]. If MTX pulmonary toxicity is confirmed, then the drug should be discontinued.

Patients should abstain from alcohol consumption while receiving MTX. The use of broad-spectrum sunscreen is advised, and sun exposure needs to be limited because of photosensitivity.

### 3.5. Drug Interactions

Although trimethoprim-sulfamethoxazole (TMP-SMX) is often used for prophylaxis against pneumocystis jiroveci pneumonia (PJP) in patients who are significantly immunocompromised, it is not recommended to be used in conjunction with MTX. Even with a small dose of MTX, this combination increases the risk of MTX side effects such as bone-marrow suppression [57,58,59,60,61]. TMP-SMX reduces renal excretion of MTX metabolites, and both TMP-SMX and MTX can cause folate deficiency that may potentiate the risk of MTX toxicity [62,63]. Alternative oral agents for PJP prophylaxis include dapsone, [64,65] atovaquone, [64], intravenous and aerosolized pentamidine, [64] or a combination of primaquine and clindamycin [64,66].

Treatment with multiple DMARDs are acceptable for the treatment of some forms of sarcoidosis [1]. However, it is recommended to avoid the concomitant use of MTX and leflunomide because they share similar side effects such that the likelihood of bone-marrow suppression and liver toxicity are significantly increased when these drugs are used concomitantly [67,68]. Drug databases or some institution’s medication ordering systems may flag non-steroidal anti-inflammatory drugs (NSAIDs) or proton pump inhibitors when concomitantly used with MTX. However, this interaction is significant only with a high dose of MTX and is usually not relevant in the case of sarcoidosis treatment (≤25 mg/week).

### 3.6. Special Situations

*Pregnancy and breastfeeding:* MTX is contraindicated in pregnancy and while breastfeeding. Women of child-bearing age should use contraception while they are using MTX. If pregnancy is planned, then MTX should be discontinued three months prior to conception for a woman [69]. In men, although MTX labeling suggests discontinuing MTX prior to attempting pregnancy, clinical data show no such risks that the continued use of MTX is conditionally recommended for men planning to father a child [69,70,71,72,73].

*Swallowing difficulties:* For patients with swallowing difficulties, a parenteral solution preparation (25 mg/mL) of MTX can be used orally with a 1:1 conversion ratio.

*Preexisting hepatic or renal conditions:* Patients with preexisting hepatic and renal conditions who receive MTX should be monitored closely, and alternative treatment agents should be considered.

*Vaccination*: Influenza vaccine and other non-live vaccines can be administered while MTX is used. Although holding MTX for two weeks after vaccination can increase the immunologic response to the vaccine, this is recommended only when the patient’s risk of a disease flare is low [35]. For live-attenuated vaccines, MTX is recommended to be held from four weeks prior to the vaccination until four weeks after vaccination [35].

### 3.7. Counseling Points for a Patient Receiving MTX

- Take MTX “one day per week”.
- Take folic acid daily seven days per week, including the day of MTX use.
- Use split dosing for weekly MTX doses of >15 mg weekly: “half of the dose in the morning then half of the dose in the evening, 12 h apart, within one day every week”.
- MTX takes up to 3~6 months of use with good adherence to reach its steady state of clinical effectiveness. Encourage the patient to take MTX as prescribed despite the drug’s initial minimal efficacy.
- Contact the healthcare provider if unexplained cough develops.
- Potential MTX side effects include birth defects, liver toxicity, bone-marrow suppression, photosensitivity (use sunscreen, wear hat and long sleeves), hair loss, mouth ulcer etc.
- Frequent blood test monitoring (CBC, serum liver, and renal function tests) is required while receiving MTX.
- Hold two doses of MTX after receiving an annual influenza vaccination to maximize vaccine efficacy if sarcoidosis symptoms are minimum and the risk of a sarcoidosis exacerbation is low.
- Contact the healthcare provider if an infection occurs, or if an invasive procedure or surgery is planned. MTX may have to be held temporarily in this instance.
- Encourage vaccination prior to initiating MTX, as vaccination is a highly effective infection mitigation strategy.
- With drug-induced immunocompromised condition, the patient is eligible for RZV, Shingrix^®^.

## 4. Leflunomide

### 4.1. Mechanism of Action

LEF is a prodrug that is converted in the gut and liver to teriflunomide, its active form. This conversion is almost complete such that its original form of LEF is practically undetectable in the serum [74,75]. LEF’s pharmacologic effectiveness is achieved by the inhibition of dihydroorotate dehydrogenase (DHODH) in the de novo synthesis of pyrimidines.

### 4.2. General Treatment Indications for Leflunomide in Sarcoidosis

LEF is regarded as a second-line agent for sarcoidosis. LEF is specifically recommended as a second-line agent for pulmonary and cardiac sarcoidosis in the European Respiratory Society (ERS) Clinical Practice Sarcoidosis Treatment Guidelines [1]. LEF has also been used successfully for skin, eye, and sinus sarcoidosis [76].

### 4.3. Dosing

The typical dose of LEF is 10 mg to 20 mg daily. Although some experts have recommended a 100 mg daily loading dose for the initial three days, this can increase the risk of drug toxicity without a substantiated clinical benefit. LEF does not require a dosage adjustment in patients with renal insufficiency.

### 4.4. Side Effects and Monitoring

Toxicities from LEF include teratogenicity, bone-marrow suppression, serious infection, reactivation of latent TB infection, interstitial lung disease, peripheral neuropathy, dermatologic reactions, hypersensitivity reactions, hepatotoxicity, alopecia, gastrointestinal symptoms (nausea, diarrhea, pain, ulcer), headache, hypertension, and dizziness.

Drug monitoring should include surveillance for signs and symptoms of the above-mentioned side effects. CBC and LFT blood tests should be performed at drug initiation, then every 2 to 4 weeks during the first 3–6 months, and then extended to every 2 to 3 months in stable patients [55].

When LEF toxicity is suspected, an accelerated elimination procedure should be performed with charcoal or cholestyramine. The oral administration of activated charcoal powder (in the form of a suspension) is 50 g every 12 h for 11 days. Cholestyramine is administered orally: 8 g three times daily for 11 days. These accelerated elimination procedures effectively block the LEF’s active metabolite, teriflunomide, from being recycled through enterohepatic pathways and force its excretion. After one day of the above regimen with cholestyramine or charcoal, teriflunomide concentration can be reduced by approximately 40% [75]. After 11 days of the accelerated elimination procedure, if the teriflunomide plasma concentration is higher than 0.02 mg/L, then the above procedure should be repeated [77]. An alternative accelerated elimination procedure of cholestyramine, 4 g every 6 h for 2 weeks has been recommended by the European Association for the Study of the Liver [78].

### 4.5. Drug Interactions

Because teriflunomide is highly protein bound (99%) [75], there is a theoretical risk that drugs used concomitantly with LEF may be displaced from their protein-bound state, leading to excessive plasma concentrations. Tolbutamide is a highly protein-bound drug where this may occur. Concomitant use of LEF and methotrexate is generally avoided because they have similar toxicities (vide supra, MTX section).

Because LEF is a CYP2C8 inhibitor, serum levels of CYP2C8 substrates such as pioglitazone, repaglinide, rosiglitazone, and selexipag may be increased in patients receiving LEF concomitantly [79,80,81]. Patients receiving LEF and warfarin concomitantly require close INR monitoring as LEF may potentiate warfarin’s effectiveness, increasing the INR [82,83]. Paradoxically, the prescribing information cautioned that the combination of LEF and warfarin may decrease peak INR by 25% without clear explanation of the mechanism [77]. We recommend that providers closely monitor the INR in patients receiving LEF and warfarin concomitantly.

### 4.6. Special Situations

*Pregnancy*: LEF is teratogenic, and therefore it is contraindicated in pregnancy. Pregnancy should be excluded prior to the initiation of LEF. Woman with reproductive potential should be advised to use effective contraception while receiving LEF. If a woman receiving LEF is found to be pregnant, an accelerated elimination procedure (vide supra) is recommended, [84]. No increased rate of birth defects has been observed with paternal exposure of LEF [85].

*Breastfeeding*: Although there is no information available concerning the concentration of LEF or its metabolites in breast milk [85], it is recommended that women not breastfeed while they are receiving the drug. There is a great potential for LEF to accumulate in breast milk because of its enterohepatic circulation. As LEF is an immunosuppressant, there is concern that the nursing baby’s immune function and immunization efficacy could be affected if their breastfeeding mother is receiving LEF.

*Renal adjustment*: Unlike MTX, LEF does not require a dose adjustment in patients with compromised renal function; therefore, LEF has a potential advantage over MTX in such patients. In dialysis patients, the terminal clearance half-life of LEF is similar to that of healthy volunteers such that there is no need for a dose adjustment [75,86].

*Hepatic adjustment*: LEF is not recommended in patients with severe hepatic insufficiency or hypoproteinemia. LEF should be discontinued if the serum ALT is >3 times of the upper limit of normal, and an accelerated elimination procedure may be indicated [77].

*Vaccination*: Influenza vaccine and other non-live vaccines can be administered while LEF is used. For live-attenuated vaccines, LEF is recommended to be held from four weeks prior until four weeks after the vaccination [35].

### 4.7. Counseling Points for a Patient Receiving LEF

- Potential side effects include birth defects, liver toxicity, bone-marrow suppression, neuropathy, blood-pressure increase, and hair loss.
- It may take up to 3~6 months of use to reach its steady state of clinical effectiveness. Encourage the patient to take LEF as prescribed with good adherence despite the LEF’s initial minimal efficacy.
- Frequent blood-test monitoring is required while receiving LEF.
- Contact the healthcare provider if an infection occurs, or if a procedure or surgery is planned that may increase the risk of infection. LEF may have to be held temporarily in this instance.
- Encourage vaccination prior to initiating LEF, as vaccination is a highly effective infection mitigation strategy.
- With drug-induced immunocompromised condition, the patient is eligible for RZV, Shingrix^®^.

## 5. Azathioprine

### 5.1. Mechanism of Action

**Figure 1:**
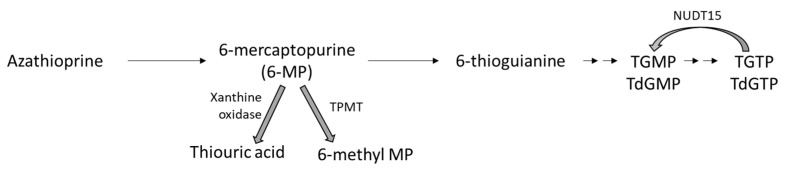
Azathioprine metabolism.

Azathioprine (AZA) is a cytotoxic immunosuppressive agent that inhibits purine nucleic acid metabolism, which ultimately suppresses cellular immunity. AZA is a prodrug of 6-mercaptopurine (6-MP), which is then further metabolized to its major active metabolite, 6-thioguanine (6-TG), which can be directly incorporated into DNA as a thioguanine nucleotide causing DNA damage (Figure 1) [87]. AZA has been used in many areas of medicine including organ transplantation, oncology, and inflammatory conditions including sarcoidosis.

TGMP—thioguanine nucleotide monophosphate;

TdGMP—thio-deoxyguanosine monophosphate;

TGTP—thioguanine nucleotide triphosphate;

TdGTP—thio-deoxyguanosine triphosphate;

TPMT—thiopurine methyltransferase;

NUDT15—nucleoside diphosphate-linked moiety X motif 15.

### 5.2. General Treatment Indications for Azathioprine in Sarcoidosis

AZA is regarded as a second-line agent for sarcoidosis. AZA is specifically recommended as a second-line agent for pulmonary, cardiac, and neurologic sarcoidosis in the European Respiratory Society (ERS) Clinical Practice Sarcoidosis Treatment Guidelines [1]. AZA has also been used successfully for eye sarcoidosis [88].

### 5.3. Dosing

For sarcoidosis, the initial AZA dose is usually 25 to 50 mg once daily, which is then increased by 50 mg every two to four weeks as clinically indicated and tolerated. The maximum daily AZA dose for the treatment of sarcoidosis has not been established but it should not exceed 250 mg/day, based on expert consensus [1]. The manufacturer has recommended to use the lower end of the therapeutic dosing range of AZA in patients with kidney impairment but did not supply specific guidance [89]. Some experts have recommended using significantly lower AZA doses in patients with renal impairment [90].

Thiopurine methyltransferase (*TPMT*) and nucleoside diphosphate-linked moiety X motif 15 (*NUDT15*) pharmacogene phenotype testing needs to be performed prior to initiation of AZA [89,91,92]. The test classifies *TPMT* and *NUDT15* phenotypes as “normal metabolizers”, “intermediate metabolizers”, or “poor metabolizers”. Poor and intermediate metabolizers are likely to have an increased concentration of active metabolites of AZA (Figure 1), which can increase drug toxicity. Prescribing information and Clinical Pharmacogenetics Implementation Consortium (CPIC) guidelines recommend not using AZA for non-oncologic conditions in patients who are poor *TPMT* and/or *NUDT15* metabolizers. Patients who are intermediate *TPMT* and/or *NUDT15* metabolizers should receive AZA dosing that is 30% to 80% less than the normal [89,92].

### 5.4. Side Effects and Monitoring

The toxic effects of AZA include bone-marrow suppression (leukopenia, anemia, thrombocytopenia), hepatic dysfunction, pancreatitis, nephrotoxicity, lymphoma, fever, gastrointestinal intolerance (nausea, vomiting, and diarrhea), skin rash, and jaundice, particularly in patients who have preexisting hepatic dysfunction, and, rarely, hepatic sinusoidal obstruction syndrome (SOS, also called veno-occlusive disease, VOD). Skin cancer has been reported to be associated with AZA [93].

Baseline CBC, renal, and hepatic-function blood tests should be obtained before initiating AZA. These tests should be monitored every two weeks while doses are being titrated and then every three months thereafter. Clinical signs and symptoms of drug toxicity should be monitored during every visit. Because individuals receiving AZA have a higher risk of non-melanoma skin cancer, close surveillance is recommended [94].

### 5.5. Drug Interactions

Xanthine oxidase inhibitors such as allopurinol and febuxostat increase the risk of AZA toxicity by inhibiting the conversion of 6-MP to inactive metabolites. This can cause the accumulation of 6-MP. Therefore, xanthine oxidase inhibitors are avoided with AZA; alternative immunosuppressives to AZA should be considered in this situation. The concomitant use of AZA with other immunosuppressant drugs such as tumor necrosis alpha inhibitors can increase the risks of infection and malignancy [94].

### 5.6. Special Situations

*Pregnancy and breastfeeding*: AZA is a pregnancy category D drug, meaning there is evidence of fetal risk [95]. The category D status was given to AZA based on studies concerning high-dose AZA treatment of leukemia [96]. However, data from clinical trials and case series suggest that anti-inflammatory doses of AZA are safe with minimal risks in pregnancy and with breastfeeding; therefore, the drug may be used in these situations [68,69,97].

Children whose mothers received AZA while they were in utero were found not to have a decrement in long-term immune function [98]. The concentration of AZA in breast milk is low enough that breastfeeding is acceptable in nursing mothers receiving this drug [99]. Men who are planning to father a child may continue receiving AZA according to the American College of Rheumatology guidelines [69,100].

*Renal impairment*: Patients receiving AZA with a CrCl <30 mL/min or receiving dialysis require a 25% to 50% dose reduction.

*Hepatic impairment*: Although AZA can cause significant hepatotoxicity, there is no standard recommendation for adjusting the AZA dose based on hepatic impairment. When AZA hepatotoxicity occurs, treatment should be paused, and a dose reduction or discontinuation of AZA should be considered. If a patient receiving AZA develops a hepatic sinusoidal obstruction syndrome (SOS; veno-occlusive disease), the drug should be permanently discontinued.

*Combination therapy of AZA with other immunosuppressants*: Because AZA, LEF, and MTX have overlapping side effects of liver toxicity, bone-marrow suppression, and increased risk of infection, patients receiving at least two of these drugs concomitantly need close observation. AZA and tumor necrosis factor alpha inhibitor (TNFi) coadministration may lead to a higher rate of malignancy compared with monotherapy [68].

*Vaccination*: Influenza vaccine and other non-live vaccines can be administered while AZA is used. For live-attenuated vaccines, AZA is recommended to be held from four weeks prior to vaccination until four weeks after vaccination [35].

### 5.7. Counseling Points for a Patient Receiving AZA

- The potential side effects of AZA include liver toxicity and bone-marrow suppression.
- It takes up to 3~6 months of use to reach its steady state of clinical effectiveness. Encourage the patient take AZA as prescribed with good adherence despite the drug’s initial minimal efficacy.
- Frequent blood-test monitoring is required while receiving AZA.
- Contact the healthcare provider if an infection occurs, or if a procedure or surgery is planned that may increase the risk of infection. AZA may have to be held temporarily in this instance.
- Encourage vaccination prior to initiating AZA, as vaccination is a highly effective infection-mitigation strategy.
- With a drug-induced immunocompromised condition, the patient is eligible for RZV, Shingrix^®^.

## 6. Mycophenolate (Mycophenolate Mofetil, Mycophenolate Sodium)

### 6.1. Mechanism of Action

Mycophenolate exhibits its immunosuppressive action by inhibiting inosine 5-monophosphate dehydrogenase (IMPDH), an enzyme involved with de novo purine nucleotides synthesis. This eventually leads to a reduction in lymphocyte proliferation, chemotaxis, and antibody production [101].

### 6.2. General Treatment Indications for Mycophenolate in Sarcoidosis

Mycophenolate is regarded as a second-line agent for sarcoidosis. Mycophenolate is specifically recommended as a second-line agent for pulmonary, cardiac, and neurologic sarcoidosis in the European Respiratory Society (ERS) Clinical Practice Sarcoidosis Treatment Guidelines [1]. MPA has also been used successfully for eye sarcoidosis [102].

### 6.3. Dosing

Mycophenolate is available in two forms: mycophenolate mofetil (MMF) and enteric coated (EC)-mycophenolate sodium. Both are hydrolyzed to the active form, mycophenolic acid (MPA). The usual daily dose of MMF is 500 mg to 3000 mg in divided doses, usually given 1500 mg twice daily. MMF 500 mg is considered equivalent to 360 mg of EC-mycophenolate sodium. MMF is converted to MPA more quickly than EC-mycophenolate sodium, and therefore it is the preferred form of the drug. It is recommended to initiate MMF at a low dose then to up-titrate to the target maintenance dose to minimize GI intolerance. There is a higher incidence of GI side effects with MMF than EC-mycophenolate sodium. Therefore, if a patient experiences GI side effects with MMF, switching to EC-mycophenolate sodium can be considered. A suspension formula (MMF, 200 mg/mL) is available that can be used in patients who have swallowing difficulties. Suspension MMF contains aspartame and is therefore contraindicated in patients with phenylketonuria.

### 6.4. Side Effects and Monitoring

Gastrointestinal complaints are the most common side effects of MMF. Often, this complication is severe enough to result in discontinuation of the drug. Patients receiving MMF are at an increased risk of infection. Due to its teratogenicity, mycophenolate preparations are contraindicated in pregnancy [101,103].

Other adverse reactions to MMF include fever, arthralgia, arthritis, myalgias, increased liver enzymes, anemia, leukopenia, thrombocytopenia, possible reactivation of hepatitis, lymphoproliferative disorders, skin cancers, hypertension, edema, dyslipidemia, renal insufficiency, and John Cunningham (JC) virus-associated progressive multifocal leukoencephalopathy (PML).

CBC, LFT, and serum renal-function tests should be performed at drug initiation, then every two to four weeks until the patient reaches a stable maintenance dose. At that point, these blood tests should be monitored every three months. For those patients with an ANC of <1.3 × 10^3^/mcL, MMF therapy should be interrupted, and the maintenance dose should be reduced, or drug discontinuation should be considered [104].

Although some studies showed benefits from monitoring MMF serum levels via therapeutic drug monitoring (TDM), optimal serum levels have not been established [105,106]. Therefore, TDM of MMF is not currently a routine practice.

### 6.5. Drug Interactions

Concomitant use of antacids containing magnesium or aluminum decreases the bioavailability of MMF because of increased gastric pH caused by antacids. It is recommended to take MMF at least 2 h after antacid use. Proton pump inhibitors (PPI), such as omeprazole, pantoprazole, and lansoprazole, may decrease MMF’s bioavailability; therefore, careful assessment of the effectiveness of MMF is warranted in this situation. Phosphate binders such as sevelamer also decrease MMF’s bioavailability. Doses of these two medications should be separated by >2 h in order to optimize the clinical effect of MMF therapy [107]. Antibiotics such as aminoglycosides, cephalosporins, fluoroquinolones, and penicillins may interfere with the enterohepatic recirculation of MMF and its metabolites, resulting in a reduction in MMF bioavailability by 30~50% [108,109]. Therefore, patients receiving these antibiotics may require higher doses of MMF [110,111]. Concomitant use of rifampin may decrease MMF bioavailability by >70% [110,111,112].

### 6.6. Special Situations

*Pregnancy*: MMF is teratogenic and is contraindicated with pregnancy. MMF is incorporated in the Risk Evaluation and Mitigation Strategy (REMS) program required by the Food and Drug Administration (FDA). This program informs doctors, nurses, pharmacists, and patients about the increased risks of taking mycophenolate during pregnancy. The American College of Rheumatology guidelines recommend that women treated with MMF who plan to conceive should stop taking MMF >6 weeks prior. For men who plan to father a child, the ACR conditionally recommends continuing MMF [69], whereas the manufacturer’s prescribing information recommends discontinuing mycophenolate at least 90 days before a trial of conception or sperm donation [104]. We recommend conducting an informed shared decision-making process with these men and considering alternative agents to MMF.

*Breastfeeding*: The manufacturer’s prescribing information states that no harmful effects have been reported in breastfeeding children based on limited clinical data. Due to lack of sufficient evidence, the American College of Rheumatology guidelines recommend against the use of MMF while breastfeeding [69].

*Renal or hepatic impairment*: The manufacturer does not recommend a MMF dosage adjustment for patients with hepatic dysfunction or renal insufficiency. However, experts have recommended limiting MMF use to a maximum dose of 1 g twice daily if the patient’s eGFR is <25 mL/min [113].

*Vaccination*: Influenza vaccine and other non-live vaccines can be administered while MMF is used. For live-attenuated vaccines, mycophenolate is recommended to be held from four weeks prior until four weeks after the vaccination [35].

### 6.7. Counseling Points for a Patient Receiving MMF

- Educate the patient concerning the potential side effects of MMF including gastrointestinal intolerance, liver toxicity, and bone-marrow suppression.
- Educate the patient that MMF takes up to 3~6 months of use to reach its steady state of clinical effectiveness. Encourage the patient take MMF as prescribed with good adherence despite the drug’s initial minimal efficacy.
- The 500 mg MMF tablets or capsules may be too big to swallow for some patients. Inform the patient that a smaller size (250 mg) capsule formulation is available. Also, suspension formulation can be considered.
- Frequent blood-test monitoring is required while receiving MMF.
- Contact the healthcare provider if an infection occurs, or if a procedure or surgery is planned that may increase the risk of infection. MMF may have to be held temporarily in this instance.
- Encourage vaccination prior to initiating MMF, as vaccination is a highly effective infection-mitigation strategy.
- With drug-induced immunocompromised condition, the patient is eligible for RZV, Shingrix^®^.

## 7. Hydroxychloroquine

### 7.1. Mechanism of Action

Hydroxychloroquine (HCQ) is an antimalarial drug with immunosuppressive activity that has been used for many inflammatory diseases including sarcoidosis. The mechanism of action of HCQ is poorly understood. HCQ is thought to increase the pH in lysosomes, causing suppression of intracellular antigen processing that subsequently leads to decreased T-lymphocyte activation and leukocyte chemotaxis [114,115].

### 7.2. General Treatment Indications for Hydroxychloroquine in Sarcoidosis

HCQ is regarded as a second-line agent for sarcoidosis. HCQ is specifically recommended as a second-line agent for pulmonary, skin, and neurologic sarcoidosis in the European Respiratory Society (ERS) Clinical Practice Sarcoidosis Treatment Guidelines [1]. Despite the European Respiratory Society (ERS), it is the authors’ experience that HCQ has inadequate potency to be effective for pulmonary sarcoidosis. Because of the risk of retinopathy from HCQ (vide infra), the drug is not recommended for the treatment of eye sarcoidosis.

### 7.3. Dosing

The usual immunosuppressive dose of HCQ is ≤5 mg/kg/day (actual body weight), with a maximum daily dose of 400 mg in two divided doses [116]. No specific adjustment is required for hepatic or renal impairment.

### 7.4. Side Effects and Monitoring

Retinopathy is a common and potentially serious toxicity of HCQ. The risk of HCQ-associated retinopathy is dependent upon the daily dose and the duration of use. At the recommended HCQ dose of ≤5 mg/kg/day, the risk of retinopathy is less than 1% during the first five years of use and increases to almost 2% over the subsequent 10 years. However, subsequently, the risk of retinopathy accelerates to 20% after 20 years of HCQ use [116]. Other HCQ side effects include cardiomyopathy [117], hemolysis in those with G6PD deficiency [118], neuropsychiatric manifestations (agitation, anxiety, depression, psychosis, and psychomotor agitation), sleep disorders (hypersomnolence, insomnia, night terrors, and nightmares) [119,120], skin toxicities (exacerbations of psoriasis and dermatitis), gastrointestinal discomfort, and QT prolongation. Hypoglycemia may occur with HCQ use in both diabetic and non-diabetic patients, especially in those receiving concomitant drugs that have hypoglycemic effects [121,122].

Baseline retinopathy screening should include a funduscopic examination within the first year of HCQ use. Visual fields and spectral domain optical coherence tomography (SD-OCT) should be performed if maculopathy is present at baseline [116]. Annual ophthalmology screening is recommended to begin after five years of HCQ use [116]. More frequent ophthalmology evaluations may be warranted if the patient is using HCQ in a high dose range (>5 mg /kg actual body weight), has a diminished estimated glomerular filtration rate (eGFR), or has a history of previous retinal disease. It is recommended that patients receiving HCQ be monitored every 6 to 12 months with the following laboratory tests: CBC, serum liver-function and renal-function tests, and serum glucose.

### 7.5. Drug Interactions

As both tamoxifen and HCQ may cause retinal toxicity, the risk of eye complication increases greatly when both drugs are used concomitantly [123]. Concomitant use of dapsone and HCQ should be prescribed with caution because of a higher risk of hemolytic reactions especially in patients with glucose-6-phosphate dehydrogenase (G6PD) deficiency or methemoglobin reductase deficiency.

With high-dose aspirin (>3 g daily) or other salicylates such as bismuth subsalicylate and salsalate, HCQ may cause hypoglycemia in both diabetic and non-diabetic patients [124,125]. HCQ can increase the blood concentration of digoxin [126]; therefore, careful monitoring is needed when these drugs are given concomitantly. Drugs that prolong the EKG QT-interval, such as ciprofloxacin, norfloxacin, sertraline, escitalopram, trazodone, and IV haloperidol, require regular EKG monitoring of the QT-interval when used concomitantly with HCQ.

### 7.6. Special Situations

Per the manufacturer’s prescribing information, HCQ dose adjustment is not required for patients with renal or hepatic insufficiency. However, the American Academy of Ophthalmology identified compromised renal function as one of the risk factors for retinopathy in long-term use patients. Therefore, some clinicians recommend to reduce the daily HCQ dose in patients with a low eGFR [127], although explicit guidance has not been established.

HCQ is safe to continue in women and men planning to have children, throughout pregnancy, and breastfeeding [69].

*Vaccination*: HCQ is considered as non-immunosuppressive by expert opinion that there are no limitations to vaccine administration [35].

### 7.7. Counseling Points for a Patient Receiving HCQ

- Educate the patient concerning potential side effects of HCQ, especially retinal toxicity, gastrointestinal intolerance, liver toxicity, and bone-marrow suppression.
- It takes up to 3~6 months of use to reach its steady state of clinical effectiveness. Encourage the patient to take HCQ as prescribed with good adherence despite the drug’s initial minimal efficacy.
- Counsel the patient that ophthalmology evaluations as surveillance for retinopathy is required while receiving HCQ.
- Educate the patient to monitor his/her body weight. Individuals weighing <80 kg (177 pounds) should receive a weight-based daily dose (not to exceed 5 mg/kg/day). Counsel the patient to report to their healthcare provider if significant weight change occurs, as HCQ dose adjustment is needed. Individuals who weigh more than 80 kg should not exceed a daily dose of 400 mg. The maximum dose of HCQ is 400 mg daily, in divided dose, regardless of the patient’s weight.
- Educate the patient that a psoriatic rash can develop or worsen while receiving HCQ, and the patient should contact their provider if such a skin reaction occurs.

## 8. Tumor Necrosis Factor Alpha Inhibitors (TNFi)

### 8.1. Mechanism of Action

Tumor necrosis factor alpha (TNFα) is a proinflammatory cytokine involved with coordination of the immune response. There is a sound rationale for this therapy in sarcoidosis, [128] because TNFa is thought to be integrally involved in the development of the sarcoid granuloma [129]. Dysregulation of TNFα production and signaling has been associated with immune-mediated disorders. Therefore, inhibition of TNFα can be an effective strategy for the treatment of sarcoidosis. The recent ERS sarcoidosis treatment guidelines recommend two tumor necrosis alpha inhibitors (TNFi), infliximab (IFX) and adalimumab (ADA), as third-line treatment options [1]. The other three marketed TNFi drugs (etanercept, certolizumab, and golimumab) either failed to demonstrate efficacy for the treatment of sarcoidosis or have not been studied [130,131].

IFX is a chimeric antibody against TNFα, containing both human and murine protein within the bioengineered antibody [132]. ADA, in comparison, is composed of 100% human protein. Fully human antibody has lower immunogenicity.

### 8.2. General Treatment Indications for Tumor Necrosis Factor Alpha Inhibitors in Sarcoidosis

IFX and ADA are regarded as third-line agents for sarcoidosis. Both IFX and ADA are specifically recommended as a third-line agent for pulmonary, skin, cardiac, and neurologic sarcoidosis in the European Respiratory Society (ERS) Clinical Practice Sarcoidosis Treatment Guidelines [1]. IFX is specifically recommended over ADA as a third-line agent for cardiac sarcoidosis. IFX and ADA are also recommended for the treatment of eye sarcoidosis [88]. IFX and ADA are particularly useful agents for the lupus pernio form of skin sarcoidosis, [133] cardiac sarcoidosis, [134] and neurosarcoidosis [135,136].

### 8.3. Dosing

The optimal dosing of TNFi for sarcoidosis is not currently standardized. Based on expert opinion, IFX and its biosimilars are usually dosed at 3–5 mg/kg via intravenous infusion at weeks 0 and 2, then once every 4–6 weeks. ADA is typically dosed at 40 mg subcutaneously every one to two weeks.

Unexpected anaphylactic reaction may occur in both IFX and ADA. Severe infusion reactions can occur with IFX that can be life threatening. Premedication with IV glucocorticoids, acetaminophen, and antihistamines are usually given prior to each IFX infusion.

### 8.4. Side Effects and Monitoring

IFX and ADA are immunosuppressive agents that increase the risk of infection including tuberculosis and hepatitis [137,138,139,140]. Prior to the initiation of IFX or ADA, the patient should have documented negative serologies for hepatitis B, hepatitis C, and negative screening for latent tuberculosis by QuantiFERON-GOLD or tuberculosis skin testing.

Both IFX and ADA can potentially develop anti-drug antibodies, but this is more common with IFX than ADA because of the chimeric design of IFX, which includes a murine protein portion. When anti-drug antibodies are formed, the TNFi treatment may become ineffective or can cause adverse reactions such as fever, rash, or bronchospasm [141]. There may be no clinical consequence from developing TNFi anti-drug antibodies; therefore, detection of anti-drug antibody is not a reason to stop the TNFi if the treatment is effective without side effects [141]. To mitigate anti-drug antibody development, concomitant use of methotrexate has been shown to be effective lowering the frequency of this complication [142].

Although the clinical data are inconsistent, TNFi drugs may increase the risk of malignancy, particularly lymphoma [143]. The American College of Rheumatology guidelines recommend that if an individual has history of solid tumor that has been cured for >5 years, then a TNFi agent can be used [55]. TNFi agents are used to treat autoimmune disorders but, paradoxically, the patient may develop autoimmune disorders by using TNFi, with symptoms such as lupus-like syndrome, skin rash, or fever [55,144,145,146,147,148,149]. TNFi drugs may cause heart failure, demyelinating disease, or worsen these conditions if those conditions were present prior to TNFi use [42,150].

If an infusion reaction occurs during IFX administration, the infusion rate may be reduced, or the infusion may be terminated if it is suspected to be an anaphylaxis event. Warning signs for anaphylaxis (hives or a choking sensation in the throat) that develop during an IFX infusion should be taken seriously and termination of the infusion should be considered.

Other side effects from TNFi include diverticulitis, autoimmune hepatitis, optic neuritis, hematologic symptoms (such as leukopenia, pancytopenia, and thrombocytopenia), headache, confusion, and tremor.

ADA injection-site reactions may occur, but in most cases these reactions have minimal consequences [151]. CBC and LFT is recommended to be obtained every six months while receiving a TNFi to monitor liver function and blood counts.

### 8.5. Drug Interactions

IFX and ADA should not be used with other immunosuppressive biologic medications or Janus Kinase inhibitors (JAKi) due to the profound immunosuppression caused by using these drugs concomitantly. Live vaccines are contraindicated during TNFi use. Drug-database interaction checkers may indicate significant drug interactions between oral DMARDs such as MTX or LEF and TNFi due to a concern of increased infection risks. However, combination therapy with a biologic and oral DMARDs is considered safe and efficacious in clinical practice with routine monitoring.

### 8.6. Special Situations

*Pregnancy:* Both IFX and ADA cross the placenta. However, they can be used during the first two trimesters of the pregnancy. At the third trimester, IFX and ADA are recommended to be discontinued to avoid significant drug concentration in neonate [69].

*Breastfeeding*: IFX and ADA are large protein molecules. It is very unlikely for these TNFi agents to reach appreciable levels in the nursing child’s blood stream via oral intake. Therefore, TNFi is considered safe to continue with breastfeeding [69].

*Compromised renal function:* No adjustment is needed for IFX or ADA because of renal dysfunction.

*Compromised hepatic function*: There are no established recommendations for adjusting IFX or ADA in patients with hepatic insufficiency.

IFX may cause elevations of serum liver enzymes, especially in patients with elevated transaminases at baseline or with metabolic dysfunction-associated steatotic liver disease (MASLD, formerly known as non-alcoholic fatty liver disease). Some experts recommend continuing IFX if the serum AST and ALT are elevated but <5 times upper limit of normal (ULN), with frequent LFT monitoring [152]. If AST and ALT are ≥5 times ULN then discontinuation of IFX may be considered [153].

*Vaccination*: Annual influenza vaccine and other non-live vaccines can be administered without interruption of IFX or ADA treatment. For live vaccines, the American College of Rheumatology recommends that IFX and ADA be held for one dose before the administration of live vaccine until four weeks after the live vaccine administration [35].

### 8.7. Counseling Points for a Patient Receiving TNFi

- Educate the patient concerning potential TNFi side effects, infections, malignancy, possible onset or worsening of congestive heart failure, or demyelinating diseases such as multiple sclerosis.
- Educate the patient that IFX or ADA may take up to three to six months to reach their steady states of clinical effectiveness. Encourage the patient take these medications as prescribed with good adherence despite the drugs initial minimal efficacy.
- ADA is a subcutaneous injection medication that can be used at home.
- IFX is administered via intravenous infusion at a clinic setting, and it typically takes several hours.
- For ADA, educate the patient on the injection technique. The first injection should be conducted in the presence of a health care professional for patient safety.
- For IFX, educate the patient that (s)he will receive pre-medications per the institution’s protocol to prevent an IFX infusion reaction.
- Inform the patient not to compensate for a missed ADA dose with an additional dose. If the patient forgets an ADA injection, the patient should perform that injection as soon as possible and consider that day as the start of a new injection cycle.
- Contact the healthcare provider if an infection occurs, or if a procedure or surgery is planned that may increase the risk of infection. The TNFi agent may have to be held temporarily in this instance.
- Three to six months may take for the medication to build up to reach its maximum effectiveness. Be patient and adhere to the medication.
- The patient should inform the healthcare provider if there is a previous history of tuberculosis, hepatitis B, or hepatitis C infection.
- Emphasize that TNFi drugs are immunosuppressants and encourage vaccine adherence to mitigate risks of vaccine-preventable diseases.
- Live vaccine is contraindicated with TNFi agents.
- With drug-induced immunocompromised conditions, the patient is eligible for RZV, Shingrix^®^.

## 9. Rituximab

### 9.1. Mechanism of Action

Rituximab (RTX) is a chimeric antibody [154] that has affinity for the CD20 receptor on subpopulations of B cells and thereby leads to their depletion via cell-mediated and complement-dependent cytotoxicity, which promotes their apoptosis [155]. CD20 is only expressed on pre-B cells and mature B cells but not on progenitor (stem) cells or plasma cells [156]. Although sarcoidosis is thought to be a T-cell mediated disease, heightened B-cell activity is also seen in active sarcoidosis, including the development of a polyclonal gammopathy [157].

### 9.2. General Treatment Indications for Rituximab in Sarcoidosis

RTX is regarded as a fourth-line agent/salvage therapy for sarcoidosis. RTX is specifically recommended as a fourth-line agent for pulmonary sarcoidosis in the European Respiratory Society (ERS) Clinical Practice Sarcoidosis Treatment Guidelines [1].

### 9.3. Dosing

The optimal dose of RTX for sarcoidosis has not been established. The usual dose of RTX for autoimmune conditions is 1 g IV at week zero and week two, and this schedule is repeated every six months. However, for sarcoidosis, the decision to repeat this schedule is iterative and based on the treatment response. Because RTX is a chimeric molecule, it has high immunogenicity and pre-medications with IV glucocorticoids, along with oral acetaminophen and antihistamine agents, are typically administered prior to infusion. No dosage adjustment of the RTX dose is needed for hepatic or renal impairment, or dialysis.

### 9.4. Side Effects and Monitoring

Boxed warnings include infusion-related reactions, severe mucocutaneous reactions, progressive multifocal leukoencephalopathy (PML), and tumor lysis syndrome. The following side effects are rare but can be severe: diverticulitis (including bowel perforation), infection-like symptoms (fever, chills), palpitations, dizziness, high or low blood pressure, chest pain, and pulmonary and hepatic toxicity [158].

Prior to RTX administration, patients should be screened serologically for hepatitis B and hepatitis C, and for latent tuberculosis infection via a QuantiFERON-GOLD assay or a tuberculin skin test. Infusion-reaction monitoring is required during RTX administration. As RTX is contraindicated during pregnancy (vide infra), women receiving the drug who have reproductive potential require monitoring of their pregnancy status. PML signs and symptoms (such as hemiparesis, visual field deficits, cognitive impairment, aphasia, and ataxia cranial nerve deficits) also need to be monitored.

### 9.5. Drug Interactions

Combined use with other immunosuppressive biologics should be avoided due to the profound immunosuppression.

### 9.6. Special Situations

*Pregnancy:* The manufacturer recommends effective contraception during therapy and for 12 months following the last RTX dose for women who have reproductive potential. The American College of Rheumatology guidelines recommend that RTX be discontinued if the patient becomes pregnant unless patient is being treated for a life-threatening or organ-threatening situation [69].

*Breastfeeding*: Breastfeeding while receiving RTX is considered acceptable [69].

Compromised renal function: No adjustment is needed.

*Compromised hepatic function*: No adjustment is needed.

*Vaccination*: Because RTX is an anti-CD20 B-cell depleting agent, the therapeutic effect of vaccines can be diminished. If a live vaccine is indicated, it should be given more than six months after the most recent RTX dose, and further RTX doses should be held for four more weeks after the live vaccine administration. Influenza vaccine and other non-live-attenuated vaccines can be administered in patients who have received RTX. It is recommended to time these vaccinations until just prior to when the next RTX dose is due, then to hold RTX for at least two weeks to enhance vaccine effectiveness [35].

### 9.7. Counseling Points for a Patient Receiving RTX

- Educate the patient concerning potential side effects of RTX.
- RTX is an intravenous infusion medication, which may take several hours to infuse.
- Contact the healthcare provider if an infection occurs while receiving RTX, or if a procedure or surgery is planned that may increase the risk of infection and follow their recommendation.
- Before you receive RTX, inform your provider if you have untreated hepatitis B, hepatitis C, tuberculosis, or previous infections that have been treated.
- Educate the patient that RTX is contraindicated in pregnancy. Pregnancy should be avoided while receiving RTX, and the patient will be monitored for pregnancy while receiving the drug.
- Emphasize that RTX is an immunosuppressant and encourage the patient to receive vaccines.
- Live vaccine is contraindicated with RTX.
- With a drug-induced immunocompromised condition, the patient is eligible for RZV, Shingrix^®^.
- Counsel the patient concerning PML symptoms such as loss of coordination, loss of language ability, memory loss, vision problems, and progressive weakness in arms and legs.

## 10. Repository Corticotropin Injection

### 10.1. Mechanism of Action

Repository corticotropin injection (RCI) is adrenocorticotropin hormone (ACTH) injected subcutaneously that activates corticotrophin receptors and melanocortin receptors (MCs). RCI activates all five subtypes of melanocortin receptors, MC1 through MC5. MC1 exists on melanocytes and macrophages and stimulates increased pigmentation. MC2 is the ACTH receptor that stimulates adrenal steroidogenesis. The side effects of RCI are therefore, not surprisingly, consistent with those caused by glucocorticoids. MC3 and MC4 are located at the CNS and spinal cord, associated with energy, food intake, and satiety control. MC5 regulates sebogenesis in lymphocytes and exocrine cells [159].

It is unclear if the mechanism of action of RCI works primarily through stimulation of corticotrophin receptors, melanocortin receptors, or both [159,160]. Stimulation of both receptors results in down regulation of several inflammatory cells involved in the formation of the sarcoid granuloma [161]. By using RCI, steroid dosages were reduced by >50% in three clinical trials such that RCI has been referred to as “a steroid sparing agent,” [162] although it is unclear if stimulation of corticotrophin receptor results in anti-inflammatory properties and side effects similar to those of glucocorticoids.

### 10.2. General Treatment Indications for Repository Corticotropin Injection in Sarcoidosis

RCI is regarded as a fourth-line agent or salvage therapy for sarcoidosis. RCI is specifically recommended as a fourth-line agent for pulmonary sarcoidosis in the European Respiratory Society (ERS) Clinical Practice Sarcoidosis Treatment Guidelines [1].

### 10.3. Dosing

The manufacturer’s prescribing information recommends “individualized dosing” for sarcoidosis, without specific guidance. Per expert opinion, the usual dose of RCI for pulmonary sarcoidosis is 40–80 units twice a week [1]. No dosage adjustment is needed for hepatic or renal impairment.

### 10.4. Side Effects and Monitoring

The side effects of RCI are similar to those from glucocorticoids: infection including hepatitis B or latent TB, adrenal suppression, electrolyte abnormalities, immunosuppression, psychiatric change (mood instability, depression, euphoria, insomnia, irritability, psychosis), fluid retention, hirsutism, hypertension, hyperglycemia, and gastrointestinal toxicities (gastritis, diverticulitis, ulcer, perforation). RCI may also cause cardiovascular complications (atrial fibrillation, heart failure, palpitations), dizziness, fatigue, headache, and malaise. An additional potential side effect from RCI is hyperpigmentation of the skin by MC1 receptor stimulation from the drug.

The monitoring of RCI use is identical to that with glucocorticoids (vide infra). Because RCI is an injectable medication, patients should be monitored for injection-site reactions.

### 10.5. Drug Interactions

RCI virtually shares the same drug interaction with glucocorticoids.

### 10.6. Special Situations

*Pregnancy and breastfeeding:* The manufacturer’s prescribing information states that the published literature on systemic corticosteroid use during pregnancy may be relevant for RCI use, suggesting similar concerns. With the current data and level of evidence, we believe that it is reasonable to consider the management of women receiving RCI during pregnancy and while breastfeeding similar to those receiving glucocorticoids.

Compromised renal function: No adjustment is needed.

*Compromised hepatic function*: No adjustment is needed.

*Vaccination*: Live and live-attenuated vaccines are contraindicated for patients receiving “immunosuppressive doses” of RCI per the manufacturer’s prescribing information. However, the cut-off of an immunosuppressive dosing level was not specified. RCI specific information regarding vaccination recommendation is scarce.

### 10.7. Counseling Points for a Patient Receiving RCI

- Educate the patient concerning potential side effects of RCI, which are practically the same as glucocorticoids plus increased pigmentation.
- RCI is a subcutaneous injection.
- RCI should be stored in a refrigerator.
- Contact the healthcare provider if an infection occurs, or if a procedure or surgery is planned that may increase the risk of infection. RCI may have to be held temporarily in this instance.
- The patient should inform the healthcare provider if there is a previous history of untreated or previously treated tuberculosis, hepatitis B, or hepatitis C infection.
- Emphasize that RTX is an immunosuppressant and encourage the patient to receive vaccines.
- Live vaccine is contraindicated in patients receiving RCI, per prescribing information.
- With drug-induced immunocompromised conditions, the patient is eligible for RZV, Shingrix^®^.

## 11. Summary

We have provided an overview of the common pharmacologic agents used for the treatment of sarcoidosis. The dosing, side effects, and monitoring of sarcoidosis drugs are summarized in Table 5. Table 6 summarizes the use of these agents in special situations. Sarcoidosis may require treatment to prevent organ-threatening or life-threatening complications of the disease. However, sarcoidosis is most commonly treated for quality-of-life [163]; in such patients, avoidance of drug side effects and drug-induced adverse events is of paramount importance. We believe that optimal use of these agents will improve sarcoidosis patient care and patient well-being.

## Figures and Tables

**Table 1:** Frequently used glucocorticoids and their comparative potency.

Compounds	Anti-Inflammatory Potency	Equivalent Dose (mg)
Cortisone	0.8	25
Hydrocortisone	1	20
Prednisolone	4	5
Prednisone	4	5
Methylprednisolone	5	4
Triamcinolone	5	4
Betamethasone	25	0.75
Dexamethasone	25	0.75

From references [2,4].

**Table 2:** Prednisone monitoring parameters.

Monitoring Parameter	Monitoring Time Frame	Reference
Body weight	Baseline, frequently.	[6]
Height	Baseline, annually.	[6,7]
Blood pressure	Baseline, frequently.	[5,6]
HbA1C	Baseline, every 3~6 months.	[6]
Blood glucose	Baseline, frequently.	[6]
CBC	Baseline, frequently.	[6]
Lipid profile	Baseline, one month after initiation of glucocorticoid therapy, then every 6–12 months.	[5,6,8]
Bone-mineral density	Baseline, every 1–2 years.	[7]
Fracture history	Baseline, then at routine follow up visits.	[6]
Joint pain	Baseline, then at routine follow up visits.	[6,9]
Infection	Baseline, then at routine follow up visits.	[5,6]
Eye exam	Baseline, then annually or as recommended by an ophthalmologist.	[5,6]
Healthy lifestyle inventory and education	Baseline documentation of patient’s lifestyle and awareness. After initial counseling, reinforce healthy lifestyle choices at routine follow up visits.	[6]
Perceived fatigue	Baseline, then at routine follow up visits.	[6,10]
Adrenal insufficiency	Measure serum cortisol or perform an ACTH stimulation test in patients with symptoms of adrenal insufficiency (or withdrawal) who have been tapered to a low dose or off corticosteroids.	[6]
Anginal symptoms (cardiovascular events)	Baseline, at routine follow up visits, educate the patient concerning these symptoms.	[6,11]

ACTH: adrenocorticotropic hormone.

**Table 3:** Examples of CYP3A4 inhibitors and inducers.

CYP450 3A4 Inhibitors		CYP450 3A4 Inducers	
INCREASED GLUCOCORTICOID EFFECTIVENESS INCREASED GLUCOCORTICOID SIDE EFFECT RISK		DECREASED GLUCOCORTICOID EFFECTIVENESS DECREASED GLUCOCORTICOID SIDE EFFECT RISK	
Moderate Effect	Strong Effect	Moderate Effect	Strong Effect
Diltiazem Verapamil Erythromycin Fluconazole Isavuconazole Cyclosporine Dronedarone	Clarithromycin Erythromycin Itraconazole Ketoconazole Voriconazole Posaconazole Ritonavir Indinavir Darunavir Nelfinavir Saquinavir	Rifapentine Rifabutin Efavirenz Bosentan	Phenobarbital Phenytoin Fosphenytoin Primidone Rifampicin Rifampin Carbamazepine Eslicarbazepine Lumacaftor Lumacaftor-ivacaftor

**Table 4:** Methotrexate dose adjustment by eGFR, adopted from Kintzel, 1995 [50].

CrCl	Methotrexate Dose
CrCl > 60 mL/min	No dose adjustment necessary.
46 ≤ CrCl < 60 mL/min	65% of normal dose.
31 ≤ CrCl < 45 mL/min	50% of normal dose.
CrCl < 30 mL/min	Avoid use.

**Table 5:** The dosing, side-effects, and contraindications of common sarcoidosis medications.

Drug	Dosage Form	Dosing	Side Effects	Contraindications per US or Canadian Label	Renal Dose Adjustment Required	Hepatic Dose Adjustment Required	PGx Dose Adjustment Required
**Prednisone** **(FDA approved as “systemic rheumatic disorders”)**	Oral	Varies. 5–30 mg daily in single or divided doses. Higher dose may be needed for severe diseases.	gastritis, nausea and other GI effects, osteoporosis, weight gain, diabetes, hypertension, fluid retention, hyperglycemia, skin atrophy, impaired wound healing, depression, mood change, adrenocortical insufficiency with inappropriate tapering, Cushing syndrome, decreased growth in children, myopathy, glaucoma, cataract, risk of infection	Herpes simplex of the eye, measles, or chickenpox (except for short term or emergency), peptic ulcer, diverticulitis, viral or bacterial infections not controlled by anti-infective treatment.	No	No	No
**Methotrexate**	Oral, SC	5-25 mg/week Split dosing for ≥15 mg for oral dosing. Split dosing in not needed for SC.	Mouth sores, bone marrow suppression, hepatotoxicity, nausea and other GI effects, hair loss, pneumonitis, photosensitivity	Pregnancy, severe hepatic insufficiency, alcohol use dialysis, chronic pleural effusion	Yes	Yes	No
**Leflunomide**	Oral	10~20 mg daily	Mouth sores, bone marrow suppression, hepatotoxicity, nausea and other GI effects, hair loss, peripheral neuropathy, increased blood pressure	Pregnancy Severe hepatic insufficiency Alcohol use	No	Yes	No
**Hydroxychloroquine**	Oral	5 mg/kg/day with a maximum of 400 mg daily given in divided doses	Retinopathy, QT prolongation, psoriasis, nausea, and other GI effects		No	No	No
**Azathioprine**	Oral	50~250 mg daily in divided doses	Bone marrow suppression, nausea and other GI effects, hepatotoxicity		No (manufacturer) Yes (experts)	No	Yes
**Mycophenolate Mofetil**	Oral tablet, capsule, and suspension	Start with 500 mg BID. Max maintenance dose 1500 mg BID. Do so slowly to avoid GI side effects	Bone marrow suppression, nausea and other GI effects, fever, arthralgia, myalgias, liver, hematological, dermatological toxicity, malignancy, hypertension, John-Cunningham (JC) virus associated Progressive Multifocal Leukoencephalopathy (PML)	Pregnancy	No	No	No
**Infliximab**	IV	Induction: 3–5 mg/kg at week 0, 2, 6. Maintenance: 3–5 mg/kg every 4–8 weeks after induction.	Serious infection, malignancy, lymphoma, heart failure, demyelinating disease, autoimmune disorder (e.g., lupus-like syndrome, fever), reactivation of latent infections such as Hepatitis B, Tuberculosis, infusion related reactions (e.g., angioedema, bronchospasm)	Severe heart failure	No	No	No
**Adalimumab**	SC	40 mg every week or every other week	Serious infection, malignancy, lymphoma, heart failure, demyelinating disease, autoimmune disorder (e.g., lupus-like syndrome, fever), reactivation of latent infections such as Hepatitis B, Tuberculosis, injection site reaction	Severe heart failure	No	No	No
**Rituximab**	IV	1 gram two weeks apart (week 0 and 2). Repeat every 6 months if clinically needed.	Serious infection, PML, reactivation of Hepatitis B, infusion related reactions (e.g., angioedema, bronchospasm), flushing, hypertension, edema, pruritis, hematologic side effects (anemia, neutropenia, hypogammaglobulinemia, leucopenia, thrombocytopenia), dyspnea	Severe, active infection, PML, hypersensitivity or anaphylactic reaction to murine proteins	No	No	No
**Repository corticotropin injection (FDA approved)**	SC	40–80 units twice weekly	Same as glucocorticoids Hyperpigmentation	Same as glucocorticoid assumed	No	No	No

PGx: Pharmacogenomics.

**Table 6:** The use of common sarcoidosis in special situations.

Drug	Safe to Administer Non-Live Vaccine, Influenza Vaccine	Safe to Administer live or Live-Attenuated Vaccine	Safe to Use during Pregnancy	Safe to Use during Breastfeeding	Drug to be Avoided for Concomitant Use	Cautions
**Prednisone** **(FDA approved as “systemic rheumatic disorders”)**	Yes	Depends on dose	Yes	Yes		Use steroid sparing agents as possible to avoid long term side effects of glucocorticoids.
**Methotrexate**	Yes, hold for 2 weeks after vaccination if possible.	Hold 4 weeks prior and 4 weeks after	No	No	Sulfamethoxazole/ trimethoprim	Folic acid supplement daily (1~4 mg daily) recommended. Leucovorin rescue in case of toxicity.
**Leflunomide**	Yes	Hold 4 weeks prior and 4 weeks after	No	No		Enterohepatic recycling occurs: Accelerated clearance process with cholestyramine or charcoal needed in case of toxicity or unplanned pregnancy
**Hydroxychloroquine**	Yes	Yes	Yes	Yes		Yearly eye exam
**Azathioprine**	Yes	Hold 4 weeks prior and 4 weeks after	Yes	Yes	Allopurinol Febuxostat	*TPMT* and/or *NUDT15* deficiency
**Mycophenolate Mofetil**	Yes	Hold 4 weeks prior and 4 weeks after	No	No		Avoid use with azathioprine (↑ risk myelosuppression) Oral suspension formulation useful for patients with swallowing issues
**Infliximab**	Yes	Hold 1 dose prior and 4 weeks after	OK 1st and 2nd trimester Hold for 3rd trimester	Yes	Other immunosuppressive biologic DMARD or Janus Kinase inhibitors	Monitor for anaphylaxis, severe infusion reaction. Consider antibody formation if efficacy wanes.
**Adalimumab**	Yes	Hold 1 dose prior and 4 weeks after	OK 1st and 2nd trimester Hold for 3rd trimester	Yes	Other immunosuppressive biologic DMARD or Janus Kinase inhibitors	
**Rituximab**	Yes	Hold 6 months prior and 4 weeks after	Discontinue at conception unless life or organ threatening condition	Yes	Other immunosuppressive biologic DMARD or Janus Kinase inhibitors	Monitor for anaphylaxis, severe infusion reaction. Consider antibody formation if efficacy wanes.
**Repository corticotropin injection (FDA approved)**	No specific recommendationSame as glucocorticoid assumed	No specific recommendation Same as glucocorticoid assumed	No specific recommendation Same as glucocorticoid assumed	No specific recommendationSame as glucocorticoid assumed	No specific recommendation Same as glucocorticoid assumed	Same as glucocorticoid assumed

DMARD: disease modifying anti-rheumatic drug.

## Data Availability

Not applicable.

## References

[1] Baughman R.P., Valeyre D., Korsten P., Mathioudakis A.G., Wuyts W.A., Wells A., Rottoli P., Nunes H., Lower E.E., Judson M.A. (2021). ERS clinical practice guidelines on treatment of sarcoidosis. Eur. Respir. J..

[2] Hupfeld C.J., Iñiguez-Lluhí J.A., Brunton L.L., Knollmann B.C. (2023). Adrenocorticotropic Hormone, Adrenal Steroids, and the Adrenal Cortex. Goodman & Gilman’s: The Pharmacological Basis of Therapeutics.

[3] Judson M.A. (2016). Corticosteroids in Sarcoidosis. Rheum. Dis. Clin. North. Am..

[4] Chrousos G.P., Vanderah T.W. (2024). Adrenocorticosteroids & Adrenocortical Antagonists. Katzung’s Basic & Clinical Pharmacology.

[5] Liu D., Ahmet A., Ward L., Krishnamoorthy P., Mandelcorn E.D., Leigh R., Brown J.P., Cohen A., Kim H. (2013). A practical guide to the monitoring and management of the complications of systemic corticosteroid therapy. Allergy Asthma Clin. Immunol..

[6] Goldman C., Judson M.A. (2020). Corticosteroid refractory sarcoidosis. Respir. Med..

[7] Humphrey M.B., Russell L., Danila M.I., Fink H.A., Guyatt G., Cannon M., Caplan L., Gore S., Grossman J., Hansen K.E. (2023). 2022 American College of Rheumatology Guideline for the Prevention and Treatment of Glucocorticoid-Induced Osteoporosis. Arthritis Rheumatol..

[8] Grundy S.M., Stone N.J., Bailey A.L., Beam C., Birtcher K.K., Blumenthal R.S., Braun L.T., de Ferranti S., Faiella-Tommasino J., Forman D.E. (2019). 2018 AHA/ACC/AACVPR/AAPA/ABC/ACPM/ADA/AGS/APhA/ASPC/NLA/PCNA Guideline on the Management of Blood Cholesterol: A Report of the American College of Cardiology/American Heart Association Task Force on Clinical Practice Guidelines. Circulation.

[9] Moghadam-Kia S., Werth V.P. (2010). Prevention and treatment of systemic glucocorticoid side effects. Int. J. Dermatol..

[10] Ahmet A., Kim H., Spier S. (2011). Adrenal suppression: A practical guide to the screening and management of this under-recognized complication of inhaled corticosteroid therapy. Allergy Asthma Clin. Immunol..

[11] Virani S.S., Newby L.K., Arnold S.V., Bittner V., Brewer L.C., Demeter S.H., Dixon D.L., Fearon W.F., Hess B., Johnson H.M. (2023). 2023 AHA/ACC/ACCP/ASPC/NLA/PCNA Guideline for the Management of Patients with Chronic Coronary Disease: A Report of the American Heart Association/American College of Cardiology Joint Committee on Clinical Practice Guidelines. Circulation.

[12] de La Red G., Mejia J.C., Cervera R., Llado A., Mensa J., Font J. (2003). Bilateral Achilles tendinitis with spontaneous rupture induced by levofloxacin in a patient with systemic sclerosis. Clin. Rheumatol..

[13] Naggar V.F., Khalil S.A., Gouda M.W. (1978). Effect of concomitant administration of magnesium trisilicate on GI absorption of dexamethasone in humans. J. Pharm. Sci..

[14] Uribe M., Casian C., Rojas S., Sierra J.G., Go V.L. (1981). Decreased bioavailability of prednisone due to antacids in patients with chronic active liver disease and in healthy volunteers. Gastroenterology.

[15] Albin H., Vincon G., Demotes-Mainard F., Begaud B., Bedjaoui A. (1984). Effect of aluminium phosphate on the bioavailability of cimetidine and prednisolone. Eur. J. Clin. Pharmacol..

[16] Tanner A.R., Caffin J.A., Halliday J.W., Powell L.W. (1979). Concurrent administration of antacids and prednisone: Effect on serum levels of prednisolone. Br. J. Clin. Pharmacol..

[17] Hazlewood K.A., Fugate S.E., Harrison D.L. (2006). Effect of oral corticosteroids on chronic warfarin therapy. Ann. Pharmacother..

[18] Bartoszek M., Brenner A.M., Szefler S.J. (1987). Prednisolone and methylprednisolone kinetics in children receiving anticonvulsant therapy. Clin. Pharmacol. Ther..

[19] McAllister W.A., Thompson P.J., Al-Habet S.M., Rogers H.J. (1983). Rifampicin reduces effectiveness and bioavailability of prednisolone. Br. Med. J..

[20] Carrie F., Roblot P., Bouquet S., Delon A., Roblot F., Becq-Giraudon B. (1994). Rifampin-induced nonresponsiveness of giant cell arteritis to prednisone treatment. Arch. Intern. Med..

[21] (1995). Effect of corticosteroids for fetal maturation on perinatal outcomes. NIH Consensus Development Panel on the Effect of Corticosteroids for Fetal Maturation on Perinatal Outcomes. JAMA.

[22] Czeizel A.E., Rockenbauer M. (1997). Population-based case-control study of teratogenic potential of corticosteroids. Teratology.

[23] Park-Wyllie L., Mazzotta P., Pastuszak A., Moretti M.E., Beique L., Hunnisett L., Friesen M.H., Jacobson S., Kasapinovic S., Chang D. (2000). Birth defects after maternal exposure to corticosteroids: Prospective cohort study and meta-analysis of epidemiological studies. Teratology.

[24] Lunghi L., Pavan B., Biondi C., Paolillo R., Valerio A., Vesce F., Patella A. (2010). Use of glucocorticoids in pregnancy. Curr. Pharm. Des..

[25] Pradat P., Robert-Gnansia E., Di Tanna G.L., Rosano A., Lisi A., Mastroiacovo P., All Contributors to the MADRE Database (2003). First trimester exposure to corticosteroids and oral clefts. Birth Defects Res. A Clin. Mol. Teratol..

[26] Ostensen M., Forger F. (2009). Management of RA medications in pregnant patients. Nat. Rev. Rheumatol..

[27] Middleton P.G., Gade E.J., Aguilera C., MacKillop L., Button B.M., Coleman C., Johnson B., Albrechtsen C., Edenborough F., Rigau D. (2020). ERS/TSANZ Task Force Statement on the management of reproduction and pregnancy in women with airways diseases. Eur. Respir. J..

[28] Boone B., Lazaroff S.M., Wheless L., Wolfe R.M., Barnado A. (2022). Rates of *Pneumocystis jirovecii* pneumonia and prophylaxis prescribing patterns in a large electronic health record cohort of patients with systemic lupus erythematosus. Semin. Arthritis Rheum..

[29] Fishman J.A., Gans H., AST Infectious Diseases Community of Practice (2019). *Pneumocystis jiroveci* in solid organ transplantation: Guidelines from the American Society of Transplantation Infectious Diseases Community of Practice. Clin. Transplant..

[30] Malpica L., Moll S. (2020). Practical approach to monitoring and prevention of infectious complications associated with systemic corticosteroids, antimetabolites, cyclosporine, and cyclophosphamide in nonmalignant hematologic diseases. Hematology Am. Soc. Hematol. Educ. Program..

[31] Mazurek G.H., Jereb J.A., Vernon A., LoBue P., Goldberg S., Castro K.G. (2010). Updated Guidelines for Using Interferon Gamma Release Assays to Detect Mycobacterium tuberculosis Infection—United States, 2010.

[32] Palacios-Gutierrez J.J., Rodriguez-Guardado A., Arias-Guillen M., Alonso-Arias R., Palacios-Penedo S., Garcia-Garcia J.M., Balbin M., Perez-Hernandez D., Sandoval-Torrientes M., Torreblanca-Gil A. (2022). Clinical and Epidemiological Correlates of Low IFN-Gamma Responses in Mitogen Tube of QuantiFERON Assay in Tuberculosis Infection Screening During the COVID-19 Pandemic: A Population-Based Marker of COVID-19 Mortality?. Arch. Bronconeumol..

[33] Hakimian S., Popov Y., Rupawala A.H., Salomon-Escoto K., Hatch S., Pellish R. (2018). The conundrum of indeterminate QuantiFERON-TB Gold results before anti-tumor necrosis factor initiation. Biologics.

[34] Kaur M., Singapura P., Kalakota N., Cruz G., Shukla R., Ahsan S., Tansel A., Thrift A.P., El-Serag H.B. (2018). Factors That Contribute to Indeterminate Results from the QuantiFERON-TB Gold In-Tube Test in Patients with Inflammatory Bowel Disease. Clin. Gastroenterol. Hepatol..

[35] Bass A.R., Chakravarty E., Akl E.A., Bingham C.O., Calabrese L., Cappelli L.C., Johnson S.R., Imundo L.F., Winthrop K.L., Arasaratnam R.J. (2023). 2022 American College of Rheumatology Guideline for Vaccinations in Patients with Rheumatic and Musculoskeletal Diseases. Arthritis Care Res..

[36] Altered Immunocompetence General Best Practice Guidelines for Immunization. https://www.cdc.gov/vaccines/hcp/acip-recs/general-recs/immunocompetence.html.

[37] Shingrix. https://www.cdc.gov/vaccines/vpd/shingles/hcp/shingrix/faqs.html.

[38] Weinblatt M.E., Coblyn J.S., Fox D.A., Fraser P.A., Holdsworth D.E., Glass D.N., Trentham D.E. (1985). Efficacy of low-dose methotrexate in rheumatoid arthritis. N. Engl. J. Med..

[39] Andersen P.A., West S.G., O’Dell J.R., Via C.S., Claypool R.G., Kotzin B.L. (1985). Weekly pulse methotrexate in rheumatoid arthritis. Clinical and immunologic effects in a randomized, double-blind study. Ann. Intern. Med..

[40] Williams H.J., Willkens R.F., Samuelson C.O., Alarcon G.S., Guttadauria M., Yarboro C., Polisson R.P., Weiner S.R., Luggen M.E., Billingsley L.M. (1985). Comparison of low-dose oral pulse methotrexate and placebo in the treatment of rheumatoid arthritis. A controlled clinical trial. Arthritis Rheum..

[41] Thompson R.N., Watts C., Edelman J., Esdaile J., Russell A.S. (1984). A controlled two-centre trial of parenteral methotrexate therapy for refractory rheumatoid arthritis. J. Rheumatol..

[42] Fraenkel L., Bathon J.M., England B.R., St Clair E.W., Arayssi T., Carandang K., Deane K.D., Genovese M., Huston K.K., Kerr G. (2021). 2021 American College of Rheumatology Guideline for the Treatment of Rheumatoid Arthritis. Arthritis Care Res..

[43] Lower E.E., Baughman R.P. (1995). Prolonged use of methotrexate for sarcoidosis. Arch. Intern. Med..

[44] Mehta S., Lightle A., Judson M.A. (2023). Renal sarcoidosis. Nephrol. Dial. Transplant..

[45] Rampon G., Henkin C., Jorge V.M., Almeida H.L. (2018). Methotrexate-induced mucositis with extra-mucosal involvement after acidental overdose. An. Bras. Dermatol..

[46] Van Ede A.E., Laan R.F., Rood M.J., Huizinga T.W., van de Laar M.A., van Denderen C.J., Westgeest T.A., Romme T.C., de Rooij D.J., Jacobs M.J. (2001). Effect of folic or folinic acid supplementation on the toxicity and efficacy of methotrexate in rheumatoid arthritis: A forty-eight week, multicenter, randomized, double-blind, placebo-controlled study. Arthritis Rheum..

[47] Pichlmeier U., Heuer K.U. (2014). Subcutaneous administration of methotrexate with a prefilled autoinjector pen results in a higher relative bioavailability compared with oral administration of methotrexate. Clin. Exp. Rheumatol..

[48] Herman R.A., Veng-Pedersen P., Hoffman J., Koehnke R., Furst D.E. (1989). Pharmacokinetics of low-dose methotrexate in rheumatoid arthritis patients. J. Pharm. Sci..

[49] Hazlewood G.S., Thorne J.C., Pope J.E., Lin D., Tin D., Boire G., Haraoui B., Hitchon C.A., Keystone E.C., Jamal S. (2016). The comparative effectiveness of oral versus subcutaneous methotrexate for the treatment of early rheumatoid arthritis. Ann. Rheum. Dis..

[50] Kintzel P.E., Dorr R.T. (1995). Anticancer drug renal toxicity and elimination: Dosing guidelines for altered renal function. Cancer Treat. Rev..

[51] Basile C., Montanaro A., Semeraro A. (2002). Should low-dose methotrexate therapy be prescribed to dialysis patients?. Nephrol. Dial. Transplant..

[52] Evans W.E., Pratt C.B. (1978). Effect of pleural effusion on high-dose methotrexate kinetics. Clin. Pharmacol. Ther..

[53] Morgan S.L., Baggott J.E., Vaughn W.H., Austin J.S., Veitch T.A., Lee J.Y., Koopman W.J., Krumdieck C.L., Alarcon G.S. (1994). Supplementation with folic acid during methotrexate therapy for rheumatoid arthritis. A double-blind, placebo-controlled trial. Ann. Intern. Med..

[54] Davis L.A., Polk B., Mann A., Wolff R.K., Kerr G.S., Reimold A.M., Cannon G.W., Mikuls T.R., Caplan L. (2014). Folic acid pathway single nucleotide polymorphisms associated with methotrexate significant adverse events in United States veterans with rheumatoid arthritis. Clin. Exp. Rheumatol..

[55] Singh J.A., Saag K.G., Bridges S.L., Akl E.A., Bannuru R.R., Sullivan M.C., Vaysbrot E., McNaughton C., Osani M., Shmerling R.H. (2016). 2015 American College of Rheumatology Guideline for the Treatment of Rheumatoid Arthritis. Arthritis Care Res.

[56] Hanoodi M., Mittal M. (2024). Methotrexate.

[57] Steuer A., Gumpel J.M. (1998). Methotrexate and trimethoprim: A fatal interaction. Br. J. Rheumatol..

[58] Jeurissen M.E., Boerbooms A.M., van de Putte L.B. (1989). Pancytopenia and methotrexate with trimethoprim-sulfamethoxazole. Ann. Intern. Med..

[59] Maricic M., Davis M., Gall E.P. (1986). Megaloblastic pancytopenia in a patient receiving concurrent methotrexate and trimethoprim-sulfamethoxazole treatment. Arthritis Rheum..

[60] Kobrinsky N.L., Ramsay N.K. (1981). Acute megaloblastic anemia induced by high-dose trimethoprim-sulfamethoxazole. Ann. Intern. Med..

[61] Thomas M.H., Gutterman L.A. (1986). Methotrexate toxicity in a patient receiving trimethoprim-sulfamethoxazole. J. Rheumatol..

[62] Ouellette S., Shah R., Razi S., Ashforth G., Wassef C. (2022). Fatal low-dose methotrexate toxicity: A case report and literature review. Dermatol. Ther..

[63] Cudmore J., Seftel M., Sisler J., Zarychanski R. (2014). Methotrexate and trimethoprim-sulfamethoxazole: Toxicity from this combination continues to occur. Can. Fam. Physician.

[64] Mantadakis E. (2020). *Pneumocystis jirovecii* Pneumonia in Children with Hematological Malignancies: Diagnosis and Approaches to Management. J. Fungi.

[65] Nazir H.F., Elshinawy M., AlRawas A., Khater D., Zadjaly S., Wali Y. (2017). Efficacy and Safety of Dapsone Versus Trimethoprim/Sulfamethoxazol for Pneumocystis Jiroveci Prophylaxis in Children with Acute Lymphoblastic Leukemia with a Background of Ethnic Neutropenia. J. Pediatr. Hematol. Oncol..

[66] Kay R., DuBois R.E. (1990). Clindamycin/primaquine therapy and secondary prophylaxis against Pneumocystis carinii pneumonia in patients with AIDS. South. Med. J..

[67] Curtis J.R., Beukelman T., Onofrei A., Cassell S., Greenberg J.D., Kavanaugh A., Reed G., Strand V., Kremer J.M. (2010). Elevated liver enzyme tests among patients with rheumatoid arthritis or psoriatic arthritis treated with methotrexate and/or leflunomide. Ann. Rheum. Dis..

[68] Biancone L., Annese V., Ardizzone S., Armuzzi A., Calabrese E., Caprioli F., Castiglione F., Comberlato M., Cottone M., Danese S. (2017). Safety of treatments for inflammatory bowel disease: Clinical practice guidelines of the Italian Group for the Study of Inflammatory Bowel Disease (IG-IBD). Dig. Liver Dis..

[69] Sammaritano L.R., Bermas B.L., Chakravarty E.E., Chambers C., Clowse M.E.B., Lockshin M.D., Marder W., Guyatt G., Branch D.W., Buyon J. (2020). 2020 American College of Rheumatology Guideline for the Management of Reproductive Health in Rheumatic and Musculoskeletal Diseases. Arthritis Rheumatol..

[70] Micu M.C., Ostensen M., Villiger P.M., Micu R., Ionescu R. (2018). Paternal exposure to antirheumatic drugs-What physicians should know: Review of the literature. Semin. Arthritis Rheum..

[71] Weber-Schoendorfer C., Hoeltzenbein M., Wacker E., Meister R., Schaefer C. (2014). No evidence for an increased risk of adverse pregnancy outcome after paternal low-dose methotrexate: An observational cohort study. Rheumatology.

[72] Beghin D., Cournot M.P., Vauzelle C., Elefant E. (2011). Paternal exposure to methotrexate and pregnancy outcomes. J. Rheumatol..

[73] Eck L.K., Jensen T.B., Mastrogiannis D., Torp-Pedersen A., Askaa B., Nielsen T.K., Poulsen H.E., Jimenez-Solem E., Andersen J.T. (2017). Risk of Adverse Pregnancy Outcome After Paternal Exposure to Methotrexate Within 90 Days Before Pregnancy. Obstet. Gynecol..

[74] Fajardo-Robledo N.S., Jacobo-Cuevas H., Perez-Guerrero E.E., Corona-Sanchez E.G., Saldana-Cruz A.M., Romero-Tejeda E.M., Rodriguez-Jimenez N.A., Totsuka-Sutto S.E., Lopez-Roa R.I., Ponce-Guarneros J.M. (2022). Therapeutic response to leflunomide in combo therapy and monotherapy is associated to serum teriflunomide (A77 1726) levels. Sci. Rep..

[75] Rozman B. (2002). Clinical pharmacokinetics of leflunomide. Clin. Pharmacokinet..

[76] Sahoo D.H., Bandyopadhyay D., Xu M., Pearson K., Parambil J.G., Lazar C.A., Chapman J.T., Culver D.A. (2011). Effectiveness and safety of leflunomide for pulmonary and extrapulmonary sarcoidosis. Eur. Respir. J..

[77] Leflunomide (Araba) Prescribing Information. https://www.accessdata.fda.gov/drugsatfda_docs/label/2011/020905s022lbl.pdf.

[78] European Association for the Study of the Liver, Clinical Practice Guideline Panel, EASL Governing Board Representative (2019). EASL Clinical Practice Guidelines: Drug-induced liver injury. J. Hepatol..

[79] Wu S., Hoang H.B., Yang J.Z., Papamatheakis D.G., Poch D.S., Alotaibi M., Lombardi S., Rodriguez C., Kim N.H., Fernandes T.M. (2022). Drug-Drug Interactions in the Management of Patients with Pulmonary Arterial Hypertension. Chest.

[80] Skerjanec A., Wang J., Maren K., Rojkjaer L. (2010). Investigation of the pharmacokinetic interactions of deferasirox, a once-daily oral iron chelator, with midazolam, rifampin, and repaglinide in healthy volunteers. J. Clin. Pharmacol..

[81] Itkonen M.K., Tornio A., Neuvonen M., Neuvonen P.J., Niemi M., Backman J.T. (2016). Clopidogrel Markedly Increases Plasma Concentrations of CYP2C8 Substrate Pioglitazone. Drug Metab. Dispos..

[82] Chonlahan J., Halloran M.A., Hammonds A. (2006). Leflunomide and warfarin interaction: Case report and review of the literature. Pharmacotherapy.

[83] Lim V., Pande I. (2002). Leflunomide can potentiate the anticoagulant effect of warfarin. BMJ.

[84] Chambers C.D., Johnson D.L., Robinson L.K., Braddock S.R., Xu R., Lopez-Jimenez J., Mirrasoul N., Salas E., Luo Y.J., Jin S. (2010). Birth outcomes in women who have taken leflunomide during pregnancy. Arthritis Rheum..

[85] Leflunomide (Arava). https://mothertobaby.org/fact-sheets/leflunomide-pregnancy/.

[86] Clinical Pharmacology and Biopharmaceuticas Reviews. https://www.accessdata.fda.gov/drugsatfda_docs/nda/98/20905_arava_biopharmr.pdf.

[87] Soares G., Rajabi-Estarabadi A., Nouri K., Nouri K. (2023). Medicines and Therapies Associated with Skin Cancer. Skin Cancer: A Comprehensive Guide.

[88] Pasadhika S., Rosenbaum J.T. (2015). Ocular Sarcoidosis. Clin. Chest Med..

[89] Azathioprine (Imuran). https://www.accessdata.fda.gov/drugsatfda_docs/label/2018/016324s039lbl.pdf.

[90] Azathioprine Lexicomp Online. Waltham, MA: UpToDate, Inc. https://online.lexi.com..

[91] Dean L., Pratt V.M., Scott S.A., Pirmohamed M., Esquivel B., Kattman B.L., Malheiro A.J. (2012). Azathioprine Therapy and TPMT and NUDT15 Genotype. Medical Genetics Summarie.

[92] Relling M.V., Schwab M., Whirl-Carrillo M., Suarez-Kurtz G., Pui C.H., Stein C.M., Moyer A.M., Evans W.E., Klein T.E., Antillon-Klussmann F.G. (2019). Clinical Pharmacogenetics Implementation Consortium Guideline for Thiopurine Dosing Based on TPMT and NUDT15 Genotypes: 2018 Update. Clin. Pharmacol. Ther..

[93] Plasmeijer E.I., Sachse M.M., Gebhardt C., Geusau A., Bouwes Bavinck J.N. (2019). Cutaneous squamous cell carcinoma (cSCC) and immunosurveillance—The impact of immunosuppression on frequency of cSCC. J. Eur. Acad. Dermatol. Venereol..

[94] Farraye F.A., Melmed G.Y., Lichtenstein G.R., Kane S.V. (2017). ACG Clinical Guideline: Preventive Care in Inflammatory Bowel Disease. Am. J. Gastroenterol..

[95] Pernia S., DeMaagd G. (2016). The New Pregnancy and Lactation Labeling Rule. Pharm. Ther..

[96] Damas O.M., Deshpande A.R., Avalos D.J., Abreu M.T. (2015). Treating Inflammatory Bowel Disease in Pregnancy: The Issues We Face Today. J. Crohns Colitis.

[97] Nguyen G.C., Seow C.H., Maxwell C., Huang V., Leung Y., Jones J., Leontiadis G.I., Tse F., Mahadevan U., van der Woude C.J. (2016). The Toronto Consensus Statements for the Management of Inflammatory Bowel Disease in Pregnancy. Gastroenterology.

[98] Angelberger S., Reinisch W., Messerschmidt A., Miehsler W., Novacek G., Vogelsang H., Dejaco C. (2011). Long-term follow-up of babies exposed to azathioprine in utero and via breastfeeding. J. Crohns Colitis.

[99] Van Assche G., Dignass A., Reinisch W., van der Woude C.J., Sturm A., De Vos M., Guslandi M., Oldenburg B., Dotan I., Marteau P. (2010). The second European evidence-based Consensus on the diagnosis and management of Crohn’s disease: Special situations. J. Crohns Colitis.

[100] Norgard B.M., Magnussen B., Larsen M.D., Friedman S. (2017). Reassuring results on birth outcomes in children fathered by men treated with azathioprine/6-mercaptopurine within 3 months before conception: A nationwide cohort study. Gut.

[101] Resman-Targoff B.H., DiPiro J.T., Yee G.C., Haines S.T., Nolin T.D., Ellingrod V.L., Posey L.M. (2023). Systemic Lupus Erythematosus. DiPiro’s Pharmacotherapy: A Pathophysiologic Approach.

[102] Daniel E., Thorne J.E., Newcomb C.W., Pujari S.S., Kacmaz R.O., Levy-Clarke G.A., Nussenblatt R.B., Rosenbaum J.T., Suhler E.B., Foster C.S. (2010). Mycophenolate mofetil for ocular inflammation. Am. J. Ophthalmol..

[103] Petri M. (2020). Pregnancy and Systemic Lupus Erythematosus. Best. Pract. Res. Clin. Obstet. Gynaecol..

[104] Mycophenolate Mofetil (Cellcept). https://www.gene.com/download/pdf/cellcept_prescribing.pdf.

[105] Mok C.C. (2017). Therapeutic monitoring of the immuno-modulating drugs in systemic lupus erythematosus. Expert Rev. Clin. Immunol..

[106] Anders H.J., Saxena R., Zhao M.H., Parodis I., Salmon J.E., Mohan C. (2020). Lupus nephritis. Nat. Rev. Dis. Primers.

[107] Pieper A.K., Buhle F., Bauer S., Mai I., Budde K., Haffner D., Neumayer H.H., Querfeld U. (2004). The effect of sevelamer on the pharmacokinetics of cyclosporin A and mycophenolate mofetil after renal transplantation. Nephrol. Dial. Transplant..

[108] Naderer O.J., Dupuis R.E., Heinzen E.L., Wiwattanawongsa K., Johnson M.W., Smith P.C. (2005). The influence of norfloxacin and metronidazole on the disposition of mycophenolate mofetil. J. Clin. Pharmacol..

[109] Borrows R., Chusney G., Loucaidou M., James A., Van Tromp J., Cairns T., Griffith M., Hakim N., McLean A., Palmer A. (2007). The magnitude and time course of changes in mycophenolic acid 12-hour predose levels during antibiotic therapy in mycophenolate mofetil-based renal transplantation. Ther. Drug. Monit..

[110] Naesens M., Kuypers D.R., Streit F., Armstrong V.W., Oellerich M., Verbeke K., Vanrenterghem Y. (2006). Rifampin induces alterations in mycophenolic acid glucuronidation and elimination: Implications for drug exposure in renal allograft recipients. Clin. Pharmacol. Ther..

[111] Annapandian V.M., Fleming D.H., Mathew B.S., John G.T. (2010). Mycophenolic acid area under the curve recovery time following rifampicin withdrawal. Indian J. Nephrol..

[112] Kuypers D.R., Verleden G., Naesens M., Vanrenterghem Y. (2005). Drug interaction between mycophenolate mofetil and rifampin: Possible induction of uridine diphosphate-glucuronosyltransferase. Clin. Pharmacol. Ther..

[113] Bergan S., Brunet M., Hesselink D.A., Johnson-Davis K.L., Kunicki P.K., Lemaitre F., Marquet P., Molinaro M., Noceti O., Pattanaik S. (2021). Personalized Therapy for Mycophenolate: Consensus Report by the International Association of Therapeutic Drug Monitoring and Clinical Toxicology. Ther Drug Monit.

[114] Lake D.F., Briggs A.D., Vanderah T.W. (2024). Immunopharmacology. Katzung’s Basic & Clinical Pharmacology.

[115] Kafaja T.K., Anwar S., Furst D.E., Vanderah T.W. (2024). Nonsteroidal Anti-Inflammatory Drugs, Disease-Modifying Antirheumatic Drugs, Nonopioid Analgesics, & Drugs Used in Gout. Katzung’s Basic & Clinical Pharmacology.

[116] Marmor M.F., Kellner U., Lai T.Y., Melles R.B., Mieler W.F., American Academy of Ophthalmology (2016). Recommendations on Screening for Chloroquine and Hydroxychloroquine Retinopathy (2016 Revision). Ophthalmology.

[117] Tonnesmann E., Stroehmann I., Kandolf R., Wolburg H., Strach K., Musshoff F., Tiemann K., Lewalter T. (2012). Cardiomyopathy caused by longterm treatment with chloroquine: A rare disease, or a rare diagnosis?. J. Rheumatol..

[118] Youngster I., Arcavi L., Schechmaster R., Akayzen Y., Popliski H., Shimonov J., Beig S., Berkovitch M. (2010). Medications and glucose-6-phosphate dehydrogenase deficiency: An evidence-based review. Drug Saf..

[119] Manzo C., Gareri P., Castagna A. (2017). Psychomotor Agitation Following Treatment with Hydroxychloroquine. Drug Saf. Case Rep..

[120] Mascolo A., Berrino P.M., Gareri P., Castagna A., Capuano A., Manzo C., Berrino L. (2018). Neuropsychiatric clinical manifestations in elderly patients treated with hydroxychloroquine: A review article. Inflammopharmacology.

[121] Shojania K., Koehler B.E., Elliott T. (1999). Hypoglycemia induced by hydroxychloroquine in a type II diabetic treated for polyarthritis. J. Rheumatol..

[122] Cansu D.U., Korkmaz C. (2008). Hypoglycaemia induced by hydroxychloroquine in a non-diabetic patient treated for RA. Rheumatology.

[123] Melles R.B., Marmor M.F. (2014). The risk of toxic retinopathy in patients on long-term hydroxychloroquine therapy. JAMA Ophthalmol..

[124] Hundal R.S., Petersen K.F., Mayerson A.B., Randhawa P.S., Inzucchi S., Shoelson S.E., Shulman G.I. (2002). Mechanism by which high-dose aspirin improves glucose metabolism in type 2 diabetes. J. Clin. Invest..

[125] Baron S.H. (1982). Salicylates as hypoglycemic agents. Diabetes Care.

[126] Leden I. (1982). Digoxin-hydroxychloroquine interaction?. Acta Med. Scand.

[127] Tett S.E. (1993). Clinical pharmacokinetics of slow-acting antirheumatic drugs. Clin. Pharmacokinet..

[128] Fehrenbach H., Zissel G., Goldmann T., Tschernig T., Vollmer E., Pabst R., Muller-Quernheim J. (2003). Alveolar macrophages are the main source for tumour necrosis factor-alpha in patients with sarcoidosis. Eur. Respir. J..

[129] Zissel G., Muller-Quernheim J. (1998). Sarcoidosis: Historical perspective and immunopathogenesis (Part I). Respir. Med..

[130] Utz J.P., Limper A.H., Kalra S., Specks U., Scott J.P., Vuk-Pavlovic Z., Schroeder D.R. (2003). Etanercept for the treatment of stage II and III progressive pulmonary sarcoidosis. Chest.

[131] Judson M.A., Baughman R.P., Costabel U., Drent M., Gibson K.F., Raghu G., Shigemitsu H., Barney J.B., Culver D.A., Hamzeh N.Y. (2014). Safety and efficacy of ustekinumab or golimumab in patients with chronic sarcoidosis. Eur. Respir. J..

[132] Jackson J.M. (2007). TNF- alpha inhibitors. Dermatol. Ther..

[133] Stagaki E., Mountford W.K., Lackland D.T., Judson M.A. (2009). The treatment of lupus pernio: Results of 116 treatment courses in 54 patients. Chest.

[134] Judson M.A., Adelstein E., Fish K.M., Feustel P.J., Yucel R., Preston S., Vancavage R., Chopra A., Steckman D.A. (2022). Outcomes of prednisone-tapering regimens for cardiac sarcoidosis: A retrospective analysis demonstrating a benefit of infliximab. Respir. Med..

[135] Chaiyanarm S., Satiraphan P., Apiraksattaykul N., Jitprapaikulsan J., Owattanapanich W., Rungjirajittranon T., Nanthasi W. (2024). Infliximab in neurosarcoidosis: A systematic review and meta-analysis. Ann. Clin. Transl. Neurol..

[136] Fritz D., Timmermans W.M.C., van Laar J.A.M., van Hagen P.M., Siepman T.A.M., van de Beek D., Brouwer M.C. (2020). Infliximab treatment in pathology-confirmed neurosarcoidosis. Neurol. Neuroimmunol Neuroinflamm.

[137] Loomba R., Liang T.J. (2017). Hepatitis B Reactivation Associated with Immune Suppressive and Biological Modifier Therapies: Current Concepts, Management Strategies, and Future Directions. Gastroenterology.

[138] Lan J.L., Chen Y.M., Hsieh T.Y., Chen Y.H., Hsieh C.W., Chen D.Y., Yang S.S. (2011). Kinetics of viral loads and risk of hepatitis B virus reactivation in hepatitis B core antibody-positive rheumatoid arthritis patients undergoing anti-tumour necrosis factor alpha therapy. Ann. Rheum. Dis..

[139] Godfrey M.S., Friedman L.N. (2019). Tuberculosis and Biologic Therapies: Anti-Tumor Necrosis Factor-α and Beyond. Clin. Chest. Med..

[140] Ai J.W., Zhang S., Ruan Q.L., Yu Y.Q., Zhang B.Y., Liu Q.H., Zhang W.H. (2015). The Risk of Tuberculosis in Patients with Rheumatoid Arthritis Treated with Tumor Necrosis Factor-α Antagonist: A Metaanalysis of Both Randomized Controlled Trials and Registry/Cohort Studies. J. Rheumatol..

[141] Atiqi S., Hooijberg F., Loeff F.C., Rispens T., Wolbink G.J. (2020). Immunogenicity of TNF-Inhibitors. Front. Immunol..

[142] Garces S., Demengeot J., Benito-Garcia E. (2013). The immunogenicity of anti-TNF therapy in immune-mediated inflammatory diseases: A systematic review of the literature with a meta-analysis. Ann. Rheum. Dis..

[143] Askling J., Baecklund E., Granath F., Geborek P., Fored M., Backlin C., Bertilsson L., Cöster L., Jacobsson L.T., Lindblad S. (2009). Anti-tumour necrosis factor therapy in rheumatoid arthritis and risk of malignant lymphomas: Relative risks and time trends in the Swedish Biologics Register. Ann. Rheum. Dis..

[144] Yanai H., Shuster D., Calabrese E., Mlynarsky L., Tumuluri S., Cohen R.D. (2013). The incidence and predictors of lupus-like reaction in patients with IBD treated with anti-TNF therapies. Inflamm. Bowel. Dis..

[145] Vermeire S., Noman M., Van Assche G., Baert F., Van Steen K., Esters N., Joossens S., Bossuyt X., Rutgeerts P. (2003). Autoimmunity associated with anti-tumor necrosis factor alpha treatment in Crohn’s disease: A prospective cohort study. Gastroenterology.

[146] Choi S.J., Ahn S.M., Oh J.S., Hong S., Lee C.K., Yoo B., Ye B.D., Yang S.K., Park S.H., Kim Y.G. (2021). Anti-tumor necrosis factor-induced lupus in patients with inflammatory bowel disease: A hospital-based cohort study from Korea. Therap. Adv. Gastroenterol..

[147] Feuerstein J.D., Cheifetz A.S. (2014). Miscellaneous adverse events with biologic agents (excludes infection and malignancy). Gastroenterol. Clin. North Am..

[148] Schiff M.H., Burmester G.R., Kent J.D., Pangan A.L., Kupper H., Fitzpatrick S.B., Donovan C. (2006). Safety analyses of adalimumab (HUMIRA) in global clinical trials and US postmarketing surveillance of patients with rheumatoid arthritis. Ann. Rheum. Dis..

[149] Burmester G.R., Panaccione R., Gordon K.B., McIlraith M.J., Lacerda A.P. (2013). Adalimumab: Long-term safety in 23,458 patients from global clinical trials in rheumatoid arthritis, juvenile idiopathic arthritis, ankylosing spondylitis, psoriatic arthritis, psoriasis and Crohn’s disease. Ann. Rheum. Dis..

[150] Kumar N., Abboud H. (2019). Iatrogenic CNS demyelination in the era of modern biologics. Mult. Scler..

[151] Murdaca G., Spano F., Puppo F. (2013). Selective TNF-alpha inhibitor-induced injection site reactions. Expert Opin. Drug. Saf..

[152] Aby E.S., Lake J.R., Vaughn B.P. (2020). The Impact of Biologics for the Management of Inflammatory Bowel Disease on Liver Enzymes. Clin. Liver Dis..

[153] Infliximab (2012). LiverTox: Clinical and Research Information on Drug-Induced Liver Injury.

[154] Reff M.E., Carner K., Chambers K.S., Chinn P.C., Leonard J.E., Raab R., Newman R.A., Hanna N., Anderson D.R. (1994). Depletion of B cells in vivo by a chimeric mouse human monoclonal antibody to CD20. Blood.

[155] Cohen S.B., Emery P., Greenwald M.W., Dougados M., Furie R.A., Genovese M.C., Keystone E.C., Loveless J.E., Burmester G.R., Cravets M.W. (2006). Rituximab for rheumatoid arthritis refractory to anti-tumor necrosis factor therapy: Results of a multicenter, randomized, double-blind, placebo-controlled, phase III trial evaluating primary efficacy and safety at twenty-four weeks. Arthritis Rheum..

[156] Sell S., Max E.E. (2001). Immunology, Immunopathology, and Immunity.

[157] Hunninghake G.W., Crystal R.G. (1981). Mechanisms of hypergammaglobulinemia in pulmonary sarcoidosis. Site of increased antibody production and role of T lymphocytes. J. Clin. Invest..

[158] Liote H., Liote F., Seroussi B., Mayaud C., Cadranel J. (2010). Rituximab-induced lung disease: A systematic literature review. Eur. Respir. J..

[159] Ramachandrappa S., Gorrigan R.J., Clark A.J., Chan L.F. (2013). The melanocortin receptors and their accessory proteins. Front. Endocrinol..

[160] Laiho L., Murray J.F. (2022). The Multifaceted Melanocortin Receptors. Endocrinology.

[161] Wang W., Guo D.Y., Lin Y.J., Tao Y.X. (2019). Melanocortin Regulation of Inflammation. Front. Endocrinol..

[162] Mirsaeidi M., Baughman R.P. (2022). Repository Corticotropin Injection for the Treatment of Pulmonary Sarcoidosis: A Narrative Review. Pulm. Ther..

[163] Judson M.A. (2017). Quality of Life in Sarcoidosis. Semin. Respir Crit. Care Med..

